# RNA-transfection of γ/δ T cells with a chimeric antigen receptor or an α/β T-cell receptor: a safer alternative to genetically engineered α/β T cells for the immunotherapy of melanoma

**DOI:** 10.1186/s12885-017-3539-3

**Published:** 2017-08-17

**Authors:** Dennis C. Harrer, Bianca Simon, Shin-ichiro Fujii, Kanako Shimizu, Ugur Uslu, Gerold Schuler, Kerstin F. Gerer, Stefanie Hoyer, Jan Dörrie, Niels Schaft

**Affiliations:** 1 Department of Dermatology, Universitätsklinikum Erlangen, Hartmannstraße 14, D-91052 Erlangen, Germany; 2 Department of Dermatology, Faculty of Medicine, Friedrich-Alexander-Universität Erlangen-Nürnberg (FAU), Erlangen, Germany; 3 Department of Genetics, Friedrich-Alexander-Universität Erlangen-Nürnberg Erlangen-Nürnberg, Erlangen, Germany; 4 Laboratory for Immunotherapy, RIKEN Center for Integrative Medical Sciences (IMS), 1-7-22 Suehiro-cho, Tsurumi-ku, Yokohama, Kanagawa 230-0045 Japan

**Keywords:** Immune evasion, Cross-reaction, MHC-downregulation, γ/δ T cell, Adoptive T-cell therapy, Chimeric antigen receptor, Melanoma, mRNA-electroporation, Zoledronate

## Abstract

**Background:**

Adoptive T-cell therapy relying on conventional T cells transduced with T-cell receptors (TCRs) or chimeric antigen receptors (CARs) has caused substantial tumor regression in several clinical trials. However, genetically engineered T cells have been associated with serious side-effects due to off-target toxicities and massive cytokine release. To obviate these concerns, we established a protocol adaptable to GMP to expand and transiently transfect γ/δ T cells with mRNA.

**Methods:**

PBMC from healthy donors were stimulated using zoledronic-acid or OKT3 to expand γ/δ T cells and bulk T cells, respectively. Additionally, CD8^+^ T cells and γ/δ T cells were MACS-isolated from PBMC and expanded with OKT3. Next, these four populations were electroporated with RNA encoding a gp100/HLA-A2-specific TCR or a CAR specific for MCSP. Thereafter, receptor expression, antigen-specific cytokine secretion, specific cytotoxicity, and killing of the endogenous γ/δ T cell-target Daudi were analyzed.

**Results:**

Using zoledronic-acid in average 6 million of γ/δ T cells with a purity of 85% were generated from one million PBMC. MACS-isolation and OKT3-mediated expansion of γ/δ T cells yielded approximately ten times less cells. OKT3-expanded and CD8^+^ MACS-isolated conventional T cells behaved correspondingly similar. All employed T cells were efficiently transfected with the TCR or the CAR. Upon respective stimulation, γ/δ T cells produced IFNγ and TNF, but little IL-2 and the zoledronic-acid expanded T cells exceeded MACS-γ/δ T cells in antigen-specific cytokine secretion. While the cytokine production of γ/δ T cells was in general lower than that of conventional T cells, specific cytotoxicity against melanoma cell lines was similar. In contrast to OKT3-expanded and MACS-CD8^+^ T cells, mock-electroporated γ/δ T cells also lysed tumor cells reflecting the γ/δ T cell-intrinsic anti-tumor activity. After transfection, γ/δ T cells were still able to kill MHC-deficient Daudi cells.

**Conclusion:**

We present a protocol adaptable to GMP for the expansion of γ/δ T cells and their subsequent RNA-transfection with tumor-specific TCRs or CARs. Given the transient receptor expression, the reduced cytokine release, and the equivalent cytotoxicity, these γ/δ T cells may represent a safer complementation to genetically engineered conventional T cells in the immunotherapy of melanoma (Exper Dermatol 26: 157, 2017, J Investig Dermatol 136: A173, 2016).

**Electronic supplementary material:**

The online version of this article (doi:10.1186/s12885-017-3539-3) contains supplementary material, which is available to authorized users.

## Background

Fueled by impressive clinical results, adoptive T-cell therapy (ACT) is gaining more and more momentum in the battle against hematologic malignancies and solid tumors. Conferring specificities for tumor antigens on T cells via antigen-receptor transfer has led to substantial tumor elimination in clinical trials [1–3].

T cells can either be engineered to express an additional α/β T-cell receptor (TCR) recognizing MHC-presented intracellular tumor antigens or they can be equipped with a chimeric antigen receptor (CAR) targeting surface antigens [4]. A CAR is usually created by merging an antibody-derived single chain variable fragment (scFv), the CD3ζ chain of a TCR and an additional co-stimulatory domain, such as CD28 [5].

On-target/off-tissue and off-target toxicities induced by receptor-engineered T cells attacking non-malignant host cells still remains a feared side-effect in adoptive T-cell therapy [6]. This can be caused by various mechanisms: i) due to the (unexpected) expression of tumor antigens on healthy tissue [7–10] or due to the cross reaction of tumor-specific receptors with host molecules [11, 12], ii) via random formation of new specificities originating from an α/β-chain-mispairing of the introduced α/β TCR with the endogenous α/β TCR, which may create a reactivity for host molecules [13, 14], and iii) owing to the possible re-activation of anergized self-reactive T cells by signaling through the transfected receptors. Another dreaded side-effect occurs as a result of the T-cell on-target over-activation culminating in a cytokine release syndrome [15, 16].

Therefore it is crucial for clinical deployment to search for mechanisms enhancing the safe application of engineered T cells. With respect to patient safety, transient receptor transfer, for instance via mRNA electroporation, is superior to permanent DNA-based transfection, as arising on-target/off-tissue and off-target toxicities are transient as well [17–20]. The successful receptor-transfer via mRNA electroporation has been well-established for many years [19, 21–23]. To obviate safety concerns emanating from a potential α/β chain mispairing with the endogenous α/β TCR, or a potential reactivation of dormant self-reactive T cells, the use of γ/δ T cells presents as an ideal solution, because the endogenous γ/δ TCR does not pair with α- or β-chains [24–26]. Equally important, activation of the endogenous γ/δ TCR will not result in autoimmunity.

Contrary to conventional CD8^+^ T cells, γ/δ T cells, which constitute up to 10% of peripheral blood T cells, do not recognize MHC-bound peptides [27, 28]. They are activated by phosphoantigens and aminobisphosphonates, such as isopentylpyrophosphate (IPP), which is upregulated in the context of metabolical disorders [29]. As tumorigenesis frequently involves disturbing the metabolism of incipient and bona fide malignant cells, γ/δ T cells play a very important role in tumor surveillance [30]. Moreover, γ/δ T cells were found to express the NKG2D receptor [31], which is also beneficial in tumor rejection, as tumors, e.g. Ewing’s sarcoma, often upregulate NKG2D ligands [32]. Importantly, owing to the MHC-independent targeting by γ/δ T cells, tumor cells cannot escape via MHC-downregulation. As for transient receptor expression using mRNA electroporation into γ/δ T cells, it can be hypothesized that the initial strike exerted by the introduced receptor may be prolonged via the endogenous receptor repertoire.

Clinical trials evaluating the intrinsic anti-tumor activity of ex vivo expanded γ/δ T cells showed promising results [33–35]. Other studies focused on boosting the anti-tumor response of γ/δ T cells in vivo with systemically administered zoledronic acid, which also proved to be successful [36]. Zoledronic acid interferes with cholesterol synthesis by blocking the enzyme farnesyl diphosphate synthase, which leads to the accumulation of the γ/δ TCR ligand IPP [37]. On top of that, γ/δ T cells, genetically engineered to express a CAR specific for CD19, exhibited anti-tumor activity in vitro and in murine leukemia models [38]. In addition, γ/δ T cells, lentivirally transfected with an α/β TCR, displayed an antigen-specific lysis of leukemic cells [39].

We have already demonstrated the feasibility of transferring a virus-specific α/β TCR into γ/δ T cells via mRNA electroporation to combat adenovirus infections ensuing hematopoietic stem cell transplantations [40] and of an NKT-TCR to render the γ/δ T cells responsive for the NKT ligand α-galactosylceramide [41]. As endogenous γ/δ TCRs do not recognize MHC molecules [27, 28], they do not evoke graft-versus-host disease after transferring γ/δ T cells into HLA-mismatched recipients [42]. With regard to adoptive T-cell therapy, this implies that γ/δ T cells can be obtained from multiple sources, including healthy donors, whose T cells are not compromised by tumor- or therapy-related immunosuppression [43, 44]. Thus, adoptive T-cell therapy could be applied to a multitude of patients, irrespective of their T-cell numbers and HLA-type.

Regarding malignant melanoma, which still ranks high among tumors with bad prognoses [45], there are various tumor antigens that can be exploited in adoptive cell therapy. The melanosomal membrane-protein glycoprotein 100 (gp100), which is enriched in melanocytes and melanoma cells [46, 47], can be targeted with an α/β TCR [19]. Besides, 90% of melanoma lesions express the surface protein melanoma-associated-chondroitin-sulfate-proteoglycan (MCSP), also known as chondroitin sulfate proteoglycan 4 (CSPG4) or high molecular weight-melanoma-associated antigen (HMW-MAA) [48], which can be attacked with CARs [49]. Of note, MCSP is also present on other tumor entities such as gliomas, [50] sarcomas, [51], and triple-negative breast cancer, [52] and on other cells within the tumor, like activated pericytes [53, 54]. Some healthy tissues, e.g. smooth muscle cells, express MCSP, but to a much lower extent [55].

In this study we aimed at establishing a protocol for the expansion and transfection of γ/δ T cells to generate clinically applicable numbers, which can easily be adapted to GMP-compliant production. We investigated the functionality of γ/δ T cells, transfected with either an α/β TCR specific for the melanoma-related antigen gp100 [19] or a second generation CAR directed against the membrane-bound melanoma antigen MCSP [49] using mRNA electroporation, in direct comparison to CD8^+^ T cells. To our knowledge, this is the first study to evaluate the functional transfer of a CAR and a melanoma-specific α/β TCR into γ/δ T cells by means of mRNA electroporation [56, 57].

## Methods

### Cells and reagents

At first healthy blood donors (aged: 19–63 years) willing to voluntarily participate in this study were selected from a donor database pre-existent in our group by availability and willingness to donate blood at the required date and time. The possibility to serve as a blood donor was publicly announced and each eligible candidate was educated and approved by a medical doctor after basic blood examinations.

Second, peripheral blood mononuclear cells (PBMC) were extracted from whole-blood, procured from those healthy donors following written informed consent and approved by the institutional review board (reference number: 166_14 B), via density centrifugation using lymphoprep (Axis-Shield, Oslo, Norway). Approximately 80% of the obtained PBMC were subjected to a two-step magnetic-activated cell sorting (MACS) according to the manufacturer’s instructions (Miltenyi, Bergisch-Gladbach, Germany) to successively isolate γ/δ^+^ T cells and CD8^+^ T cells. Purified T cells and remaining PBMC were resuspended at 10^6^ cells/ml before expansion (see below) in R10 medium consisting of RPMI 1640 (Lonza, Basel, Switzerland) supplemented with 2 mM L-glutamine (Lonza), 100 IU/ml penicillin (Lonza), 100 mg/ml streptomycin (Lonza), 10% (*v*/v) heat-inactivated fetal calf serum (PAA, GE healthcare, Piscataway, NY, USA), 2 mM HEPES (PAA, GE healthcare), and 2 mM β-mercaptoethanol (Gibco, Life Technologies, Carlsbad, CA, USA). Note that the fetal calf serum would have to be replaced by human serum for full GMP compliance.

Target cell lines incorporated the TxB cell hybridoma *T2.A1* (HLA-A2^+^, gp100^−^, MCSP^−^; kind gift from Prof. Dr. Schulz, Nuremberg), and the melanoma cell lines *Mel526* (HLA-A2^+^, gp100^+^, MCSP^+^; kind gift from Prof. Dr. Hinrich Abken, Köln) and *A375M* (HLA-A2^+^, gp100^−^, MCSP^+^; kind gift from Dr. Aarnoudse, Leiden, Netherlands; ATCC CRL-3223). The human lymphoma cell line Daudi (ATCC CCL-213) was a kind gift from Dr. Manfred Smetak (Nuremberg). Target cells were cultured in R10 medium, before undergoing co-incubation with effector cells. *Mel526* and *A375M* were additionally pulsed with the HLA-A2-restricted peptide gp100_280–288_ (YLEPGPVTA) as previously described [58] where indicated. Peptide-pulsing was performed in DC-medium, which consists of RPMI 1640 (Lonza), 1% human serum (Sigma-Aldrich, Taufkirchen, Germany)(heat-inactivated, 30 min, 56 °C), 2 mM L-glutamine (Lonza), and 0.04% 20 mg/l gentamycin (Lonza).

### T-cell expansion

PBMC were directly activated (on the day of isolation) with a single dose of zoledronic acid (Zoledronsäure HEXAL®_,_ HEXAL, Germany) applied at a final concentration of 5 μM [59] or with 0.1 μg/ml anti-CD3 antibody OKT3 (Orthoclone OKT3; Jannsen-Cilag, Neuss, Germany). Concomitantly, MACS-isolated γ/δ^+^ T cells and CD8^+^ T cells were stimulated with 0.1 μg/ml OKT3 directly after isolation (on the same day). Ensuing T-cell expansion was performed in alignment with a GMP-compliant protocol devised by our group [60]. In brief, 1000 IU/ml interleukin-2 (Proleukin; Novartis, Nuremberg, Germany) was administered on days 0, 2, 3, 5, and 7. On day 3, cells were counted and re-adjusted to 0.2 × 10^6^ cells/ml by adding fresh medium. On day 7, the total cell culture volume was first doubled, and subsequently split by transferring half of the volume to a second culture flask. After 10–11 days, cells were counted and prepared for further experiments.

### Flow cytometric analyses of phenotypic parameters

A FITC-labeled pan TCR γ/δ IgG1 antibody (Thermo Fisher Scientific, USA) was used in combination with PE-conjugated anti-CD3 IgG1 (ImmunoTools, Germany) and PE-conjugated anti-CD8 IgG1 (BD Biosciences, USA) antibodies to analyze the cellular composition of cell populations pre- and post-expansion. Unstained and isotype-stained cells served as controls. Immunofluorescence was measured using a FACScan cytofluorometer (BD Biosciences, Heidelberg, Germany) equipped with CellQuest software (BD Biosciences). Data were analyzed using FCS Express 5 (De Novo Software, USA).

### In vitro transcription of RNA

A TCR specific for the HLA-A2-restricted peptide consisting of amino acids 280–288 (YLEPGPVTA) of the melanosomal glycoprotein 100 (gp100) and a second generation CAR (MCSP_HL_-CD28/CD3ζ-CAR) directed against MCSP (melanoma-associated chondroitin sulfate proteoglycan) were used for transfer into T cells. The molecular compositions of both receptors were specified previously [19, 51]. In vitro transcription of receptor-encoding mRNA was performed with T7 RNA polymerase (mMESSAGE mMACHINE T7 Ultra kit; Life Technologies, Carlsbad, CA, USA) according to the manufacturer’s instructions. Afterwards, RNA was purified on RNeasy columns (Qiagen GmbH, Hilden, Germany) according to the manufacturer’s instructions. RNA quality was assessed by agarose gel electrophoresis.

### RNA electroporation

RNA transfection was executed as detailed elsewhere [19, 61]. In short, following 10–11 days of expansion, T cells were washed in OptiMem (Life technologies, Carlsbad, CA, USA,) resuspended at 10^6^ cells/ml and transferred to 4-mm gap electroporation cuvettes (Biolabproducts GmbH, Bebensee, Germany). Cells were either mock-electroporated (no RNA), transfected with 15 μg of RNA coding for the gp100/A2-specific TCR α- and β-chains [19], with 15 μg RNA encoding the MCSP-specific CAR (MCSP_HL_ CD28-CD3ζ), or with 15 μg RNA encoding the carcinoembryonic antigen (CEA)-specific CAR (CEA CD28-CD3ζ) [62] using a Gene Pulser Xcell (Bio-Rad, Hercules, CA, USA) at 500 V (square wave pulse) for 5 ms. After transfection, T cells were immediately transferred to R10 medium.

### Surface expression of transfected receptors

Surface expression of the introduced receptors was analyzed flow-cytometrically 1, 2, 4, 6, and 20 h (Fig. 2c-f), and 4, 24, 48, 72, 96, and 120 h (Fig. 2g-j) after electroporation. The TCR was stained with a PE-conjugated anti-Vbeta 14 antibody (clone CAS 1.1.3; Beckman Coulter, Immunotech, Marseille, France) and the CAR was stained with a PE-conjugated goat-F(ab’)2 anti-human IgG antibody (Southern Biotech, Birmingham, AL, USA) directed against the extracellular *IgG1 CH2CH3* CAR-domain. Additionally, an aliquot of TCR-transfected cells was cryopreserved one day after electroporation. After thawing, a PE-conjugated MHC-Dextramer HLA-A*0201/YLEPGPVTV was employed to detect the transferred TCR.

Immunofluorescence was measured using the FACScan cytofluorometer (BD Biosciences) equipped with CellQuest software (BD Biosciences). Data were analyzed using FCS Express 5.

### Cytokine secretion

Cytokine secretion by transfected T cells was assayed as described before [58]. In short, 4 h after electroporation T cells were harvested and stimulated over night at a 1:1 ratio with UV-irradiated (0.005 J/cm^2^) human lymphoma cell lines *T2.A1* and *Daudi*, as well as with human melanoma cell lines *Mel526* and *A375M*, which were employed either loaded with the HLA-A2-restricted peptide gp100_280–288_ at a concentration of 10 μg/ml or nonloaded. Cytokine concentrations in the supernatants were determined using the Th1/Th2 Cytometric Bead Array Kit II (BD Biosciences) according to the manufacturer’s instructions. Immunofluorescence was detected using the FACS Canto II (BD Biosciences, Franklin Lakes, NJ, USA) equipped with FACSDiva software (BD Biosciences). Data were analyzed using FCS Express 5.

Additionally, intracellular cytokine staining was performed as follows: 4 h after electroporation, T cells were incubated over night at a 1:1 ratio with T2.A1 cells and gp100-pulsed A375M cells. Cytokine secretion was blocked by adding Brefeldin A and Monensin (eBioscience, San Diego, CA, USA) one hour after starting the co-incubation. For FACS analysis, cells were labeled with the viability marker LIVE/DEAD (Invitrogen, Carlsbad, CA, USA). Thereafter, fixation and permeabilization was performed using the Foxp3/Transcription Factor Fixation/Permeabilization Concentrate and Diluent kit (eBioscience, San Diego, USA). Next, APC-H7-CD3 (BD Biosciences), PE-TCRγ/δ (Life Technologies, Carlsbad, CA, USA), APC-IL-2 (BD Biosciences), PE-Cy7-TNF (BD Biosciences), and Alexa Fluor 700 IFNγ (BD Biosciences) antibodies were employed to stain surface markers as well as intracellular cytokines. Immunofluorescence was detected using the LSRFortessa (BD Biosciences) equipped with FACSDiva software. The gating strategy was applied as follows: After doublet exclusion, a gate was drawn around lymphocytes and finally dead cells were excluded by viability staining. Data were analyzed using FCS Express 5.

### Cytotoxicity

Specific cytotoxicity of transfected T cells was examined with a standard 4–6 h ^51^chromium-release assay 24 h after electroporation, as previously described [58]. In brief, human tumor cell lines *T2.A1*, *Mel526*, *A375M,* and *Daudi* were labeled with 20 μCi of Na_2_^51^CrO^4^/10^6^ cells (PerkinElmer, Waltham, MA, USA) for 1 h. An aliquot of A375M cells was additionally loaded with the HLA-A2-restricted peptide gp100_280–288_ at a concentration of 5 μg/ml for 1 h. Subsequently, target cells were transferred to 96-well plates and co-incubated with effector cells at decrementing effector to target ratios. Chromium-release in the supernatants was measured after 4 h with the Wallac 1450 MicroBeta plus Scintillation Counter (Wallac, Turku, Finnland). Percentage of cytolysis was calculated as follows: [(measured release – background release)]/[(maximum release – background release)] × 100%.

### Figure preparation and statistical analysis

Graphs were created and statistical analysis was performed using GraphPad Prism, Version 6 (GraphPad Software, USA). *P*-values were analyzed using the Students t-test. * indicates *p* ≤ 0.05, ** indicates *p* ≤ 0.01, and *** indicates *p* ≤ 0.001.

## Results

### Zoledronic acid-mediated expansion of γ/δ T cells directly from PBMC is more efficient than expanding MACS-isolated γ/δ T cells

**Fig. 1:**
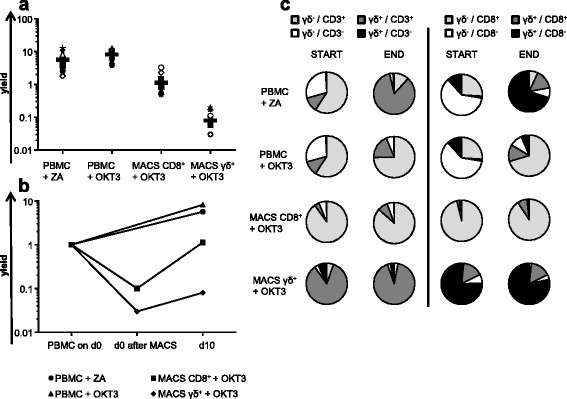
Zoledronic acid can be used to expand γ/δ T cells in a GMP-adaptable fashion. Healthy donor-derived peripheral blood mononuclear cells (PBMC) were expanded by either zoledronic acid (ZA) or OKT3 as described in the Methods section. An aliquot was subjected to magnetic-activated cell sorting (MACS) yielding γ/δ^+^ and CD8^+^ T cells. These cells were concomitantly expanded in the presence of OKT3 for 10–11 days. On days 0, 2, 3, 5, and 7, IL-2 was added. On day 3, cells were counted and re-adjusted to 0.2 × 10^6^ cells per ml by adding fresh medium. On day 7, the total cell culture volume was doubled using fresh medium and half of the total volume was transferred to a second culture flask. **a** + **b** After 10–11 days, cells were counted and the yield was calculated by dividing final cell count by the number of input PBMC. **a** Average yield of 11 to 14 independent experiments, each depicted as a different symbol, is indicated as bar. **b** Average yield of ZA-expanded γ/δ T cells (*circles*), OKT3-activated PBMC (*triangles*), and MACS-sorted CD8^+^ T cells (*squares*) and OKT3-activated-MACS γ/δ T cells (*diamonds*) – before and after OKT3-mediated expansion. Data are presented as means of 11 to 14 independent experiments. **c** Composition of cell populations before and after purification and expansion. γ/δ T cells are selectively expanded from donor-derived PBMC by ZA. Shown are double stainings for γ/δ and CD3 (*left-hand side*), and γ/δ and CD8 (*right-hand side*). Exact percentages of the different populations are summarized in Additional file 1: Table S1. Data are presented as means of 7 to 10 independent experiments

Effective application of γ/δ T cells in adoptive T-cell therapy requires ex vivo expansion to sufficient numbers. Therefore, healthy-donor PBMC were isolated from whole blood. Subsequently, one part of these PBMC was expanded directly by adding zoledronic acid (ZA), one part was expanded directly by adding anti-CD3 antibody (OKT3), and the other two parts were subjected to magnetic-activated cell sorting (MACS)-bead isolation for either CD8^+^ T cells or γ/δ^+^ T cells directly on day 0, after which OKT3 was added. This resulted in four cell populations originating from the same PBMC population. All cell populations were propagated for 10–11 days in accordance with a GMP-compliant T-cell-expansion protocol developed in our group [60]. Thereafter, expansion efficiency was deduced from the average yield (Fig. 1a and b), calculated by dividing the final cell count by the number of input PBMC. ZA-stimulation induced expansion to 6 times the original PBMC in average, which was similar to the eight-fold increase exhibited by OKT3-stimulated PBMC (Fig. 1a and b) containing also other CD3^+^ T cells (Additional file 1: Table S1). Proliferation of MACS-isolated γ/δ T cells did not approximate starting numbers of input PBMC (yield <1), whereas proliferation of MACS-isolated CD8^+^ T cells restored the cell count of input PBMC (yield approximately 1)(Fig. 1a and b). In general, the yield resulting from directly stimulating PBMC was far superior to expanding MACS-isolated cells (Fig. 1a and b). To analyze the cellular composition before and after expansion, a flow cytometric analysis for γ/δ TCR and either CD3 or CD8 expression was performed (Fig. 1c and Additional file 1: Table S1). ZA mediated a selective outgrowth of γ/δ^+^/CD3^+^ cells to 84.8%, which equals the proportion of γ/δ^+^/CD3^+^ cells obtained by purifying γ/δ T cells via magnetic-activated cell sorting before stimulation with OKT3 (Fig. 1c and Additional file 1: Table S1). After activating MACS-isolated γ/δ T cells with OKT3, the percentage of γ/δ^+^/CD3^+^ cells increased to 91.65% (Fig. 1c and Additional file 1: Table S1). Applying OKT3 to PBMC did only induce a small outgrowth of γ/δ^+^/CD3^+^ cells, and preferentially expanded γ/δ^−^/CD8^+^ cells (Fig. 1c and Additional file 1: Table S1). MACS-isolation of CD8^+^ cells resulted in a highly pure CD8^+^ cell population (> 95%), which was approximately maintained during OKT3 stimulation, resulting in the expansion of all CD8^+^ T cells including γ/δ^+^/CD8^+^ T cells (Fig. 1c and Additional file 1: Table S1).

Collectively, expanding γ/δ T cells directly from PBMC with ZA leads to higher numbers of cells and is therefore more efficient with respect to clinical application than stimulating MACS-isolated γ/δ T cells.

### γ/δ T cells can be transfected with a gp100/HLA-A2-specific TCR and an MCSP-specific CAR using mRNA electroporation

**Fig. 2:**
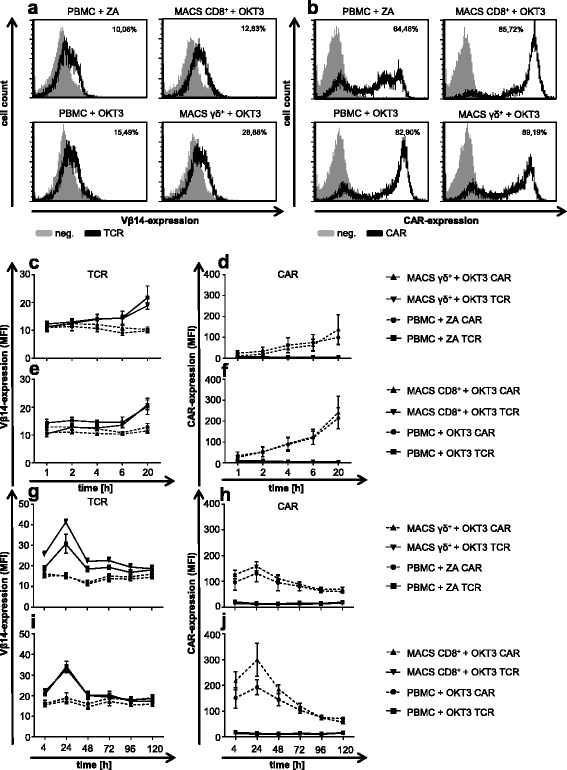
Expression of a gp100/HLA-A2-specific TCR and a MCSP-specific CAR by different T-cell populations after electroporation. ZA-activated PBMC, OKT3-activated PBMC, OKT3-stimulated MACS-isolated CD8^+^ T cells, and OKT3-stimulated MACS-isolated γ/δ T cells were expanded as described for Fig. 1. After 10–11 days, these cells were electroporated with RNA coding for the gp100/HLA-A2-specific TCR or with RNA encoding the MCSP-specific CAR. **a** + **b** TCR expression **a** was detected using an anti-Vbeta14 antibody and CAR expression levels **b** were analyzed using an anti-IgG1 antibody (*black lines*). TCR-transfected T cells served as negative controls (neg.; *filled grey* histograms) for the CAR staining and vice versa. Presented histograms are representatives out of four to seven independent experiments. **c**-**f** Short interval expression kinetics of transfected receptors on different cell populations. **g**-**j** Long interval expression kinetics of transfected receptors on different cell populations. Staining for TCR (**c** + **e** + **g** + **i**) and CAR (**d** + **f** + **h** + **j**) expression was performed at indicated time-points after electroporation using abovementioned antibodies. TCR-transfected T cells served as negative controls for CAR staining and vice versa. The percentage of positive cells is indicated. Data represent geometric means ± SEM from 4 to 7 independent experiments

To determine whether the γ/δ T cells can be reprogrammed with specificities for melanoma antigens, these cells were electroporated with RNA coding for a gp100/HLA-A2-specific TCR [19], and with RNA encoding a MCSP-specific CAR (MCSP_HL_ CD28-CD3ζ) [49]. The successful transfection of ZA-expanded γ/δ T cells and MACS-isolated, expanded γ/δ T cells was confirmed using an anti-Vbeta 14 antibody for the TCR (Fig. 2a) and an anti-IgG1 antibody for the CAR (Fig. 2b). MACS-isolated CD8^+^ and T cells resulting from OKT3-stimulated PBMC were also successfully transfected (Fig. 2a and b). The obtained percentages for the TCR expression are somewhat misleading; the complete peak of the histogram has shifted to the right, indicating that most T cells do express the TCRβ chain stained with the anti-Vβ14 antibody. Unfortunately, this PE-labeled antibody is not particularly bright and a clearer shift could not be obtained, as it was already observed before [19]. To corroborate on the TCR-transfection of aforementioned populations, a MHC-Dextramer (HLA-A*0201/YLEPGPVTV) staining was performed (Additional file 1: Figure S1). Indeed, the MACS CD8^+^ + OKT3 and PBMC + OKT3 T cell populations which were transfected with the gp100/HLA-A2-specific TCR showed a clear staining with the Dextramer (Additional file 1: Figure S1). However, since the MHC-Dextramer binding is influenced by the CD8 molecule, which is only present on a small percentage of γ/δ T cells (Additional file 1: Table S1), a direct comparison between γ/δ T cells and CD8^+^ T cells cannot be made. Moreover, receptor expression kinetics was determined by time-course experiments, which involved successive cell-surface staining with the above mentioned antibodies. To determine the time-point at which the receptor-transfected T cells can be used in a suggested clinical setting, a time-course with short intervals was performed (Fig. 2c-f). TCR expression was observed after 4–6 h and had further increased at 20 h after transfection (Fig. 2c and e). All assayed cell types displayed a similar behavior regarding TCR expression over time. Concerning CAR expression kinetics, a constant increase was observed from one hour after until 20 h after electroporation (Fig. 2d and f). To determine the duration of receptor expression, a time-course with long intervals was performed (Fig. 2g-j, and Additional file 1: Table S2). The expression was highest at 24 h and declined after that, with a minor expression still detectable at 120 h after electroporation (Fig. 2g-j; statistics in Additional file 1: Table S2).

Taken together, these results demonstrate that mRNA electroporation is an appropriate procedure to transfer melanoma-specific antigen receptors into γ/δ T cells.

### Receptor-transfected γ/δ T cells exhibit an antigen-specific cytokine secretion in response to human melanoma cells

**Fig. 3:**
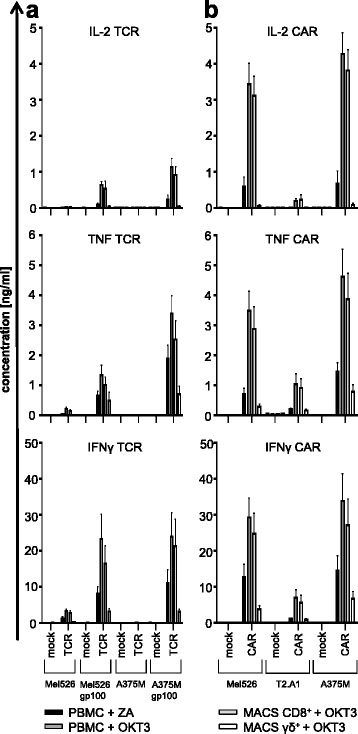
Antigen-specific cytokine production of receptor-transfected γ/δ T cells in comparison to CD8^+^ T cells. The different T-cell conditions were expanded as mentioned above (Fig. 1). After that, these cells were electroporated with RNA coding for a gp100/HLA-A2-specific TCR **a** or with RNA encoding a MCSP-specific CAR **b**. T cells electroporated without RNA (mock) were used as controls. Four hours after electroporation, T cells were co-incubated over night with human tumor cell lines at a 1:1 ratio. Induced cytokine secretion was quantified in the supernatant with a Cytometric Bead Array (CBA). Concentrations of IL-2, TNF, and IFNγ are depicted [ng/ml]; please note the different scales. Data represent means ± SEM from 7 to 10 independent experiments. *P*-values calculated by unpaired Student’s t-test are presented in Additional file 1: Table S3. **a** The cytokine secretion of gp100 TCR-RNA-transfected T cells was characterized in response to co-culture with the, either unloaded or gp100-peptide (HLA-A2 restricted) pulsed, human melanoma cell lines Mel526 and A375M. **b** The quantity of cytokines produced by MSCP CAR-RNA-transfected T cells was measured ensuing co-culture with the human lymphoma cell line T2.A1, and the melanoma cell lines Mel526 and A375M

Next, we examined whether the introduced receptors were functional. To assess the antigen-specific secretory capacity of ZA-expanded γ/δ T cells and MACS-isolated γ/δ T cells, both were transfected with the gp100/A2-specific TCR or the MCSP-specific CAR and cytokine secretion profiles were characterized. The receptor-transfected cells were stimulated with the human melanoma cell lines Mel526 and A375M, which were either targeted unloaded or, due to the low (Mel526) or absent (A375M) endogenous gp100 expression [63], loaded with an HLA-A2-restricted gp100 peptide. These cytokine profiles were compared to those of receptor-transfected MACS-isolated CD8^+^ T cells, as well as to bulk T cells, amplified from PBMC by OKT3. γ/δ T cells expressing the gp100-specific TCR produced interferon gamma (IFNγ) and tumor necrosis factor (TNF) upon cognate antigen encounter (Fig. 3a and Additional file 1: Table S3). γ/δ T cells, equipped with a MCSP-specific CAR released TNF, IFNγ, and some interleukin-2 (IL-2) in response to the above mentioned melanoma cells (Fig. 3b and Additional file 1: Table S3). These results demonstrate the functionality of the gp100-specific receptor and the MCSP-specific CAR in γ/δ T cells. Regarding quantity, both TCR- and CAR-transfected ZA-expanded γ/δ T cells exceeded the cytokine secretion of receptor-transfected MACS-isolated γ/δ T cells (Fig. 3a and b, and Additional file 1: Table S3). However, receptor-transfected MACS-isolated CD8^+^ cells and OKT3-stimulated PBMC produced more cytokines after stimulation with the cognate antigen (Fig. 3a and b, and Additional file 1: Table S3). Of note, CAR transfection of MACS-isolated CD8^+^ T cells and OKT3-stimulated PBMC and subsequent stimulation with target cells induced a background cytokine production with T2.A1 cells, serving as negative controls, which was not detected in γ/δ T cells (Fig. 3b and Additional file 1: Table S3). Cells electroporated without RNA (mock) did not display any substantial cytokine release (Fig. 3a, b, and Additional file 1: Table S3).

**Fig. 4:**
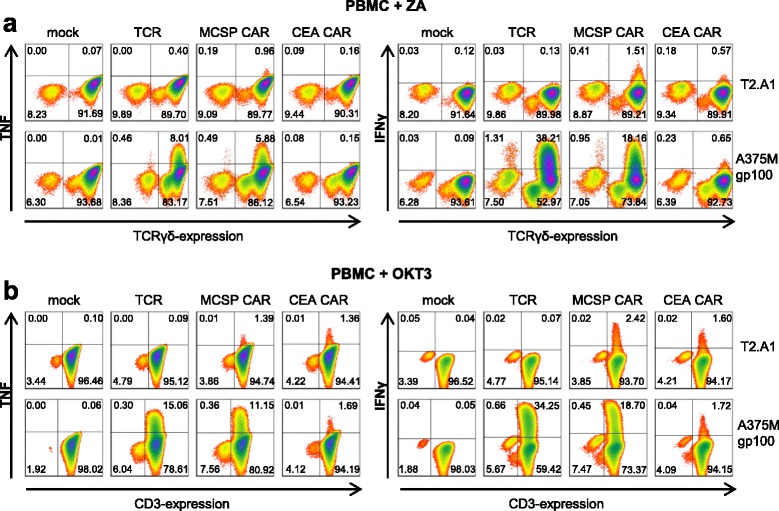
Antigen-specific cytokine production of receptor-transfected γ/δ T cells and bulk T cells. PBMC were activated with ZA or OKT3 and expanded as mentioned above (Fig. 1). After that, these cells were electroporated with RNA coding for a gp100/HLA-A2-specific TCR or with RNA encoding a MCSP-specific CAR. T cells electroporated without RNA (mock) or with RNA coding for a CEA-specific CAR were used as negative controls. Four hours after electroporation, T cells were co-incubated over night with human tumor cell lines at a 1:1 ratio. Induced cytokine production was examined using intracellular cytokine staining. Double stainings for γ/δ (**a**) or CD3 (**b**) and TNF (**a** + **b**; *left panels*) or IFNγ (**a** + **b**; *right panels*) are depicted and percent positives are shown. Presented plots are representatives out of 4 independent experiments. **a** TNF (*left panels*) and IFNγ (*right panels*) production of transfected ZA-expanded γ/δ T cells was characterized in response to co-culture with T2.A1 cells (*upper rows*) or gp100-pulsed A375M melanoma cells (*lower rows*). **b** TNF (*left panels*) and IFNγ (*right panels*) production of transfected OKT3-expanded bulk T cells was characterized in response to co-culture with T2.A1 cells (*upper row*) or gp100-pulsed A375M melanoma cells (*lower row*)

To corroborate that the secretory capacity of receptor-transfected ZA-expanded PBMC is, in fact, predominantly exerted by γ/δ T cells, these cells were depleted of γ/δ TCR-negative cells via untouched negative selection on day 10 after activation (Additional file 1: Figure S2a) and electroporated the following day. Stimulation with the aforementioned target cells resulted in an antigen-specific secretion of TNF and IFNγ by TCR- and CAR-transfected γ/δ T cells (Additional file 1: Figure S2b and c, and Additional file 1: Table S4). In terms of quantity, depleting γ/δ^−^ cells caused an approximately 50% decline in antigen-specific cytokine secretion, which is similar to the quantity secreted by receptor-transfected MACS-isolated γ/δ T cells (compare Fig. 3 and Additional file 1: Figure S2b and c). To formally prove that the receptor-transfected γ/δ T cells produced the cytokines, we conducted intracellular cytokine stainings with the TCR- and CAR-transfected OKT3- and ZA-expanded PBMC, which were stimulated with T2.A1 and A375M cells, the latter loaded with the gp100 peptide (Fig. 4). Here we included another, different CAR, specific for carcinoembryonic antigen (CEA) as additional negative control, as this antigen is not expressed on the target cells. Indeed, we observed that the γ/δ TCR-positive population transfected with the gp100-specific TCR or the MCSP-specific CAR produced IFNγ and TNF upon cognate antigen encounter (Fig. 4a). Like in the depletion experiment (Additional file 1: Figure S2), also in the intracellular FACS staining no antigen-specific IL-2 production by the receptor-transfected γ/δ T cells was observed (data not shown). When looking at the CD3-positive population, also an antigen-specific cytokine production was observed (Fig. 4b). Mock-electroporated T cells did not respond to the target cells, and only a very minor percentage of T cells electroporated with a control CAR specific for CEA showed cytokine production (Fig. 4).

In summary, these results proof that γ/δ T cells produced cytokines antigen-specifically after a melanoma-specific receptor was introduced. Furthermore, a substantial part of the secretory activity exhibited by ZA-expanded T cells can be traced back to γ/δ T cells. In addition, the data show that a substantial portion of the cells was functionally transfected with the TCR, revealing that the Vβ14-staining shown in Fig. 1 was indeed underestimating the transfection efficiency.

### Receptor-transfected γ/δ T cells specifically lyse melanoma cells

**Fig. 5:**
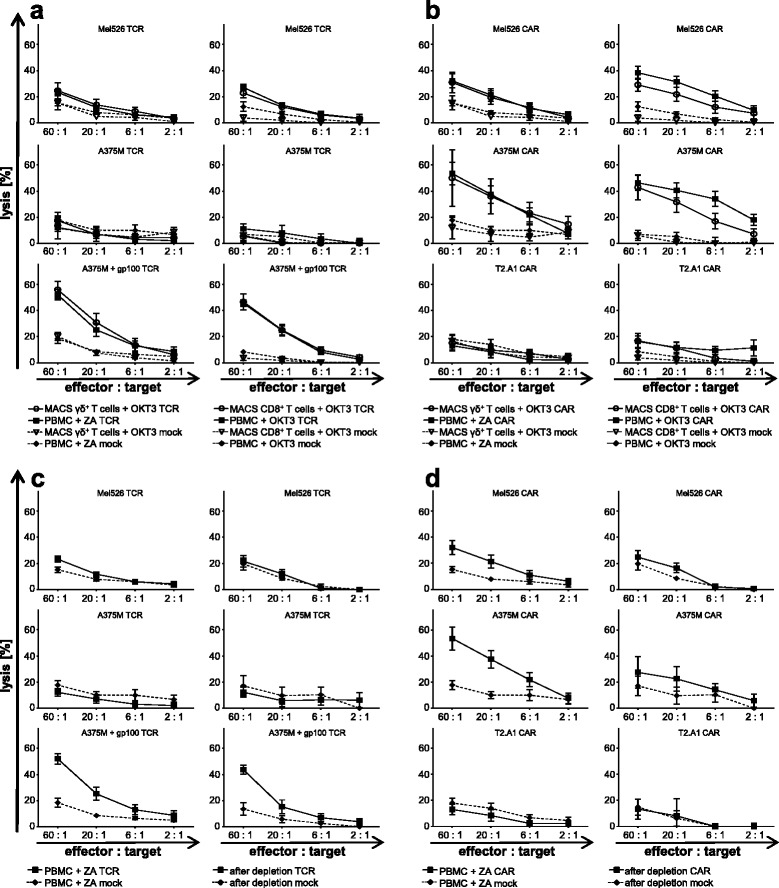
Antigen-specific lysis by receptor-transfected γ/δ T cells of human melanoma cells. ZA-activated γ/δ T cells (*left panels; closed symbols*), OKT3-activated PBMC (**a** + **b**; *right panels; closed symbols*), OKT3-stimulated MACS-isolated CD8^+^ T cells (**a** + **b**; *right panels; open symbols*), and OKT3-stimulated MACS-isolated γ/δ T cells (**a** + **b**; *left panels; open symbols*) were obtained as described above (Fig. 1). Following 10 days of expansion, untouched γ/δ T cells were isolated from an aliquot of stimulated cells via negative selection using the TCR γ/δ T Cell Isolation Kit (**c + d**, *right panels*). Cells were electroporated as described above (Fig. 2). Mock-electroporated T cells (**a** + **b**; *dotted lines*; mock) served as controls. After over-night culture, the cytolytic capacity of receptor-transfected T cells towards human tumor cell lines was examined at indicated effector to target ratios in a standard 4–6 h chromium release assay. The percentage of lysed cells was calculated. Data are presented as means ± SEM derived from 4 to 7 independent experiments (**a** + **b**) or 3 independent experiments (**c** + **d**), each performed in technical triplicate. *P*-values calculated by unpaired Student’s t-test are presented in Additional file 1: Table S5 for **a** + **b** and in Additional file 1: Table S6 for **c** + **d**. **a** + **c** gp100 TCR-transfected T cells were co-incubated with human melanoma cell lines Mel525, A375M, and gp100-peptide pulsed A375M. **b** + **d** The human lymphoma cell line T2.A1 and the human melanoma cell lines Mel526 and A375M served as targets for MCSP CAR-RNA-transfected T cells

Antigen-dependent tumor elimination is an essential prerequisite for adoptive T-cell therapy, therefore the cytolytic ability of receptor-transfected ZA-expanded γ/δ T cells, MACS-isolated γ/δ T cells, MACS-isolated CD8^+^ T cells, and PBMC stimulated with OKT3 was compared in a standard 4–6 h chromium release assay at indicated effector to target ratios (Fig. 5a and b, and Additional file 1: Table S5). Target cells included the melanoma cell lines Mel526 and A375M, which were employed either unloaded or loaded with the HLA-A2-restricted gp100 peptide. Upon gp100-TCR transfer, ZA-expanded γ/δ T cells and MACS-isolated γ/δ T cells displayed a strong antigen-specific cytotoxicity towards peptide-loaded A375M, which equaled the killing by simultaneously tested non-γ/δ T cells (Fig. 5a and Additional file 1: Table S5). This underpins our hypothesis that equipping γ/δ T cells with a gp100-specific TCR confers a strong melanoma-specific cytolytic capacity on these cells. Target cells expressing lower levels of gp100 (Mel526) were less efficiently recognized and lysed (Fig. 5a and Additional file 1: Table S5). Moreover, ZA-expanded γ/δ T cells and MACS-isolated γ/δ T cells, both transfected with the MCSP-specific CAR, eliminated human melanoma cell lines Mel526 and A375M to a similar extent as non-γ/δ T cells (Fig. 5b and Additional file 1: Table S5). This reflects the functional activity of the MCSP-CAR in γ/δ T cells. Importantly, CAR-transfected cells displayed background lysis against the MCSP-negative T2.A1 cell line (Fig. 5b and Additional file 1: Table S5). Interestingly, mock-electroporated γ/δ T cells revealed a slightly enhanced activity towards Mel526 and A375M, which was not observed in mock-electroporated MACS-isolated CD8 T cells (Fig. 5a-d, and Additional file 1: Table S5 and Additional file 1: Table S6). Mock electroporation of OKT3-expanded PBMC also exhibited an elevated lysis of target cells, which might be attributed to the presence of some γ/δ T cells, which were also propagated by OKT3 (Fig. 5a and b and Additional file 1: Table S5 and Additional file 1: Table S1). This underscores our hypothesis that receptor-transfected γ/δ T cells lyse melanoma cells using both the transferred receptors and their intrinsic anti-tumor activity.

To determine to which extent the cytolytic capacity of ZA-expanded T cells can be ascribed to γ/δ T cells, the expansion was executed for 10 days and the γ/δ TCR negative cells were depleted via untouched negative selection. After 24 h of recovery, the TCR and CAR were transfected and on the following day the cytolytic capacity of ZA-expanded cells and negatively selected γ/δ T cells (Additional file 1: Figure S2a) was examined in a standard 4–6 h chromium release assay. gp100-TCR-transfected γ/δ T cells retained their pronounced cytolytic activity against peptide-loaded A375M melanoma cells, even though on a slightly decreased level (Fig. 5c and Additional file 1: Table S6). Negatively selected γ/δ T cells expressing the MCSP-specific CAR displayed a decrease of approximately 50% in tumor lysis (Fig. 5d and Additional file 1: Table S6). The intrinsic anti-tumor effect of ZA-expanded γ/δ T cells, manifesting itself in an elevated lysis by mock-electroporated cells post depletion, was still operative and similar to non-depleted γ/δ T cells.

In sum, these results demonstrate that receptor-transfected γ/δ T cells are capable of specifically lysing human melanoma cells after being equipped with a gp100/A2-specific TCR or a MCSP-specific CAR. Furthermore, γ/δ T cells were found to target melanoma cells with their intrinsic anti-tumor activity. Finally, these data demonstrate that the tumor-specific lysis of ZA-expanded PBMC is exerted, at least to a large part, by the γ/δ T cells.

### γ/δ T cells retain their intrinsic cytotoxic activity towards Daudi cells after electroporation

**Fig. 6:**
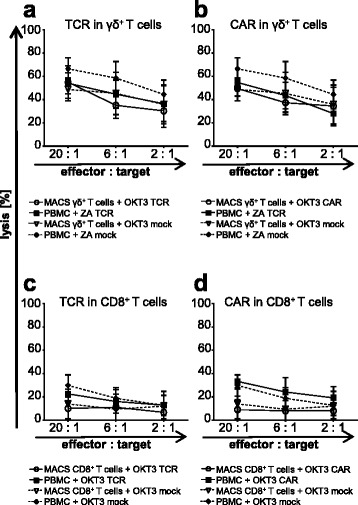
γ/δ T cells maintain their intrinsic cytolytic activity against Daudi cells after receptor transfection. ZA-activated γ/δ T cells (**a + b**; *closed symbols*), OKT3-stimulated MACS-isolated γ/δ T cells (**a** + **b**; *open symbols*), OKT3-activated PBMC (**c** + **d**; *closed symbols*), and OKT3-stimulated MACS-isolated CD8^+^ T cells (**c + d**; *open symbols*) were expanded and electroporated as described above (Figs. 1 and 2). Mock-electroporated T cells (*dotted lines*; mock) served as controls**.** After over-night culture, the cytolytic capacity of TCR- (**a** + **c**) and CAR- (**b** + **d**) transfected T cells towards Daudi cells was examined at indicated effector to target ratios in a standard 4–6 h chromium release assay. The percentage of lysed cells was calculated. Data are presented as means ± SEM derived from 3 to 5 independent experiments, each performed in technical triplicate. *P*-values calculated by unpaired Student’s t-test are presented in Additional file 1: Table S7

Transfer of a gp100/A2-specific TCR enables antigen-specific targeting of melanoma cells. Some tumors, however, downregulate antigen presentation, thus rendering themselves invisible to TCR-mediated immunosurveillance [64]. γ/δ T cells are capable of killing cells with low MHC expression via disengagement of inhibitory receptors, such as NKG2A [65]. This capacity is reflected in the ability of γ/δ T cells to lyse β2-microglobuline-deficient Daudi cells, which do not express MHC complexes on their surface [66]. Therefore, the lytic capacity of TCR- and CAR-transfected ZA-expanded γ/δ T cells and MACS-isolated γ/δ T cells against Daudi cells was ascertained in a standard 4–6 h chromium release assay and was compared to OKT3-activated PBMC and MACS-isolated CD8^+^ T cells after TCR- and CAR-transfection. All γ/δ T cells, receptor-transfected or mock-electroporated, showed a strong cytolytic activity against Daudi cells (Fig. 6a and b, and Additional file 1: Table S7). This indicates that these cells are still able to kill targets without MHC expression after electroporation. This may counteract MHC downregulation as a possible route of immune evasion. Contrary to MACS-isolated CD8^+^ T cells, which did not exert any substantial lytic effect on Daudi cells, OKT3-stimulated PBMC killed Daudi cells to a moderate extent (Fig. 6c and d, and Additional file 1: Table S7). This can be rationalized with the concurrent expansion of γ/δ T cells from PBMC using OKT3.

In summary, γ/δ T cells maintained their killing capacity against MHC-negative Daudi cells even after transfection with a gp100/A2-specific TCR and a MCSP-specific CAR, whereas γ/δ negative T cells failed to kill Daudi cells.

## Discussion

In the current study we present a novel strategy for the adoptive T-cell therapy of melanoma. So far, the majority of adoptively transferred T cells has been based on conventional CD4^+^ or CD8^+^ T cells, which were transduced with DNA encoding either an additional α/β TCR or a CAR specific for tumor-antigens [1–4]. To mitigate concerns about permanent off-target toxicities [7–12] arising from the use of stably transduced α/β T cells, e.g., α/β chain mispairing [13, 14], we explored the transient RNA-based introduction of melanoma-specific antigen receptors into γ/δ T cells. This is the first study to report on the GMP-compliant transfer of a TCR and a CAR into γ/δ T cells using mRNA electroporation for the use in adoptive T-cell therapy against melanoma.

We electroporated γ/δ T cells with mRNA encoding a gp100/HLA-A2-specific α/β TCR [19] or a CAR specific for MCSP [49]. Over the course of many years, mRNA electroporation has proven to be a reliable procedure to equip T cells with TCRs or CARs for a limited time-span [19, 21–23]. Contrary to the permanent DNA-based transfection, electroporating receptor-encoding RNA is far safer, as potential off-target toxicities cease after the receptor expression has diminished. Therefore, RNA-transfection represents an ideal avenue for testing engineered T cells with respect to auto-reactivity [19, 21]. This additional level of safety comes at the expense of a timely limited anti-tumor activity, which ceases while the receptor expression declines. This issue can be addressed by a variety of mechanisms: i) Repetitive injections of transfected T cells, which favors the use of γ/δ T cells, as they can be obtained in large quantities from healthy donors. ii) Direct application of receptor-transfected T cells into tumor lesions minimizes the time required for locating and accessing the tumor and maximizes the period, in which the tumor can be antigen-specifically targeted with the transiently-expressed receptors. As for melanoma, direct injection into cutaneous lesions is conceivable. But some locations of tumors are ill-suited for direct injection, such as the liver, which is a prime location of melanoma metastases [67]. To maximize the concentration of effector T cells in the liver, we propose a direct application of transfected T cells into the metastases via a transarterial catheter. Regarding the burden this intervention imposes on the patient, up to two successive intrahepatic injections of transfected T cells would be feasible. This procedure of directly attacking liver metastases has been successfully applied in clinical practice for delivering radioactive isotopes to the tumor [68, 69]. iii) The initial strike exerted by the transiently introduced receptors may be prolonged by the intrinsic anti-tumor activity of γ/δ T cells. One of the major traits of these cells is their ability to kill tumor cells via their endogenous receptor repertoire recognizing metabolic disorder and stress ligands [29, 31]. This was experimentally validated by the detection of an increased lysis of melanoma cells by mock-electroporated γ/δ T cells in contrast to MACS-isolated CD8^+^ T cells. Moreover, we hypothesize that the first strike exerted by the transfected receptors will further disrupt the metabolic homeostasis of tumor cells and result in an enhanced recognition by γ/δ T cells and thus maintain the anti-tumor response. In sum, these mechanisms provide attractive strategies to compensate the temporally confined anti-tumor activity associated with RNA-transfection.

Our functional characterizations concerning cytokine secretion and cytotoxic capacity toward melanoma cell lines showed that receptor-transfer via mRNA electroporation has conferred an additional specificity for melanoma antigens on γ/δ T cells. Hence, these cells can attack melanoma cells in a variety of ways: i) The α/β TCR allows for a MHC-dependent targeting of intracellularly localized tumor antigens, such as gp100, which constitute the majority of tumor antigens [19, 70]. In general, α/β TCRs can mediate a destabilization of the tumor micro-environment by killing tumor-associated myeloid-derived stromal cells, which cross-present phagocytosed tumor antigens [71, 72]. ii) The CAR targets surface antigens, e.g. MCSP, in an MHC-independent manner [49]. Apart from being expressed on tumor cells, MCSP is also present on the surface of activated pericytes involved in tumor neo-angiogenesis [53, 54].This enables a direct targeting of tumor cells coupled with an indirect targeting via the destruction of tumor-associated vasculature. iii) As described above, the endogenous receptor repertoire of γ/δ T cells [29, 31], recognizes tumors in an MHC-independent fashion [27, 28] and may become more and more active as the tumor cells and their micro-environment is pressurized by the transfected receptors, potentially supported by a radio-chemotherapy prior to the adoptive cell transfer [73]. MHC-independent targeting is also beneficial in case of MHC-downregulation or antigen-loss, which represent major strategies of immune evasion [64, 74]. iv) MHC-downregulation is further addressed by the γ/δ T-cell-inherent capacity to eliminate MHC-deficient cells, such as Daudi cells [66]. In sum, γ/δ T cells can mount a tumor-specific attack via introduced and endogenous receptors and sustain this attack even after the tumor has lost its antigen or shut down antigen presentation.

By using zoledronic acid (ZA) instead of anti-CD3 antibody in conjunction with a GMP-compliant expansion protocol, originally designed for conventional T cells [60], the selective expansion of γ/δ T cells to clinically applicable numbers was achieved. In theory, these receptor-transfected γ/δ T cells could also be obtained from a healthy donor and infused into an HLA-mismatched patient (in an immunosuppressed setting), since γ/δ T cells are not allo-reactive [27, 28, 42]. After 10–11 days of expansion, a moderate percentage of propagated cells remained CD3^+^/γδ^−^. In contrast to the predominating γ/δ T cells, these contaminating T cells might lead to graft-vs-host disease [75] in such an allogenic setting. Therefore, further adjustments are required to minimize the percentage of γ/δ^−^ cells, to employ this therapy not only in autologous settings but also in allogenic settings. Possible options involve extending the expansion course [59] and administering repeated doses of ZA. Contrary to previous work on ZA-expanded γ/δ T cells [59, 76], we additionally performed functional testing after depleting the γ/δ^−^ cells post expansion via MACS-untouched isolation to validate that the functional activity originates from γ/δ T cells. We detected a considerable reduction in cytokine production and cytolytic capacity. This would imply that a part of secreted cytokines and displayed cytotoxicity is exerted by the remaining γ/δ^−^ cells. This, however, collides with the fact that the lytic capacity of purified MACS CD8^+^ T cells and OKT3-stimulated PBMC is similar to ZA-expanded γ/δ T cells. Thus, we think that the depletion procedure per se impaired the functionality of the receptor transfected cells by interfering with cell physiology. In addition, the use of antibody-coated magnetic beads and their alike is difficult to perform under GMP compliance. Therefore, the future focus should be on rendering depletion unnecessary by refining the process of ZA-expansion to reduce the percentage of contaminating conventional T cells.

Unlike the majority of previous studies on γ/δ T cells [38, 39, 77], we evaluated our results in direct comparison to conventional α/β T cells, notably CD8^+^ T cells, which represent the current gold standard in adoptive T-cell therapy. After receptor transfer, ZA-expanded γ/δ T cells exhibited a similar lytic capacity compared to CD8^+^ T cells but a lower cytokine production. In contrast to ZA-expanded CAR-transfected γ/δ T cells, CAR transfection induced an unspecific background cytokine secretion in conventional α/β T cells, manifesting itself against the MCSP^−^ cell line T2.A1. This poses a potential danger of off-target toxicities and reframes the possible superiority of ZA-expanded γ/δ T cells. Generally, the fact that the lytic capacity of γ/δ T cells clearly exceeded their capacity to produce cytokines may also add to the safety of this approach, because a severe and potentially deadly [16] side-effect of adoptive T-cell transfer is the so called cytokine release syndrome [15], which is caused by a massive systemic release of pro-inflammatory cytokines from the transplanted cells.

The overall anti-tumor effect of adoptive T-cell therapy does not only rely on the individual functionality of the infused cells, but also on maintaining a sufficient concentration of transfected T cells in the blood [78, 79]. Due to their absent allo-reactivity [27, 28, 42], γ/δ T cells could be obtained from healthy donors, if used in immunodeficient recipients. Therefore, injecting higher numbers of γ/δ T cells in shorter intervals over a longer period of time may be possible and is a clear advantage over conventional T cells, which have to be obtained in an autologous fashion from the patient [80]. The latter may be difficult anyway, since T cells of tumor-bearing patients, especially at an advanced stage, often exhibit dysfunctionalities and exhaustion phenotypes, reflected in the upregulation of inhibitory receptors, such as PD-1 [81–84] and in functional impairments [85, 86].

## Conclusion

In summary, we developed a protocol based on ZA-expansion and mRNA electroporation to transfect γ/δ T cells with a gp100/HLA-A2-specific TCR and an MCSP-specific CAR to enlarge the therapeutic options for the adoptive T-cell therapy of melanoma. These RNA-transfected T cells responded to melanoma cells with antigen-specific tumor cell lysis to a similar extent as conventional T cells. In addition, γ/δ T cells were found to retain their intrinsic anti-tumor activity and their ability to lyse MHC-deficient cells. These characteristics in conjunction with the absent allo-reactivity and the reduced chance to cause auto-immunity qualify γ/δ T cells as a promising complementation to conventional T cells in the fight against cancer.

## Additional files

Additional file 1: Table S1. Cellular composition of different cell populations pre and post expansion based on double stainings for γ/δ and CD3, and for γ/δ and CD8. **Figure S1.** Expression of a gp100/HLA-A2-specific TCR by different T-cell populations after electroporation. Zoledronic acid (ZA)-activated PBMC, OKT3-activated PBMC, OKT3-stimulated MACS-isolated CD8^+^ T cells, and OKT3-stimulated MACS-isolated γ/δ T cells were expanded as described in Fig. 1. After 10–11 days, these cells were electroporated with RNA coding for the gp100/HLA-A2-specific TCR or with RNA encoding the MCSP-specific CAR. After receptor transfer, T cells were rested for one day and subsequently cryopreserved. After thawing, TCR expression was detected using a PE-conjugated MHC-Dextramer HLA-A*0201/YLEPGPVTV (black lines). CAR-transfected T cells served as negative controls (neg.; filled grey histograms). Presented histograms are representatives out of three independent experiments. **Table S2.** Statistical analysis corresponding to Fig. 2g-j. **Table S3.** Statistical analysis corresponding to Fig. 3. **Figure S2.** Zoledronic acid-expanded γ/δ T cells retain their cytokine secretory capacity after depletion of γ/δ^−^ cells. **a** Donor-derived PBMC were expanded with ZA (PBMC + ZA) as explained above (Fig. 1). Following 10 days of expansion, untouched γ/δ T cells were isolated from an aliquot of stimulated cells via negative selection using the TCR γ/δ T Cell Isolation Kit (after depletion). Subsequently, a γ/δ and CD3 double staining was employed to flow-cytometrically verify the successful depletion procedure. **b + c** On day 11, negatively isolated γ/δ T cells (after depletion, grey bars) and the remaining ZA-expanded T cells (black bars) were electroporated with RNA coding for the gp100/A2-specific TCR (**b**) or with RNA encoding the MCSP-specific CAR (**c**). T cells electroporated without RNA (mock) served as controls (**b + c**). Antigen-specific cytokine secretion was determined as described above (Fig. 3). Data represent means ± SEM from 4 independent experiments. *P* values calculated by unpaired Student’s t test are presented in **Table S4.** **Table S4.** Statistical analysis corresponding to **Figure S2.** b, c. **Table S5.** Statistical analysis corresponding to Fig. 5a, b. **Table S6.** Statistical analysis corresponding to Fig. 5c, d. **Table S7.** Statistical analysis corresponding to Fig. 6. (PDF 291 kb)

## Abbreviations

CAR: Chimeric antigen receptor
CBA: Cytometric Bead Array
FACS: Fluorescence-activated cell sorting
GMP: Good manufacturing practice
gp100: Glycoprotein 100
MACS: Magnetic-activated cell sorting
MCSP: Melanoma-associated chondroitin sulfate proteoglycan
MHC: Major histocompatibility complex
PBMC: Peripheral blood mononuclear cells
TCR: T-cell receptor
ZA: Zoledronic acid
