# Supplementary material for: RNA-transfection of γ/δ T cells with a chimeric antigen receptor or an α/β T-cell receptor: a safer alternative to genetically engineered α/β T cells for the immunotherapy of melanoma
Source: BMC Cancer. 2017 Aug 17;17:551. doi: 10.1186/s12885-017-3539-3 (PMC5561563; doi:10.1186/s12885-017-3539-3)
Supplement: Supplementary file 1 — Cellular composition of different cell populations pre and post expansion based on double stainings for γ/δ and CD3, and for γ/δ and CD8. Figure S1. Expression of a gp100/HLA-A2-specific TCR by different T-cell populations after electroporation. Zoledronic acid (ZA)-activated PBMC, OKT3-activated PBMC, OKT3-stimulated MACS-isolated CD8+ T cells, and OKT3-stimulated MACS-isolated γ/δ T cells were expanded as described in Fig. 1. After 10–11 days, these cells were electroporated with RNA coding for the gp100/HLA-A2-specific TCR or with RNA encoding the MCSP-specific CAR. After receptor transfer, T cells were rested for one day and subsequently cryopreserved. After thawing, TCR expression was detected using a PE-conjugated MHC-Dextramer HLA-A*0201/YLEPGPVTV (black lines). CAR-transfected T cells served as negative controls (neg.; filled grey histograms). Presented histograms are representatives out of three independent experiments. Table S2. Statistical analysis corresponding to Fig. 2g-j. Table S3. Statistical analysis corresponding to Fig. 3. Figure S2. Zoledronic acid-expanded γ/δ T cells retain their cytokine secretory capacity after depletion of γ/δ− cells. a Donor-derived PBMC were expanded with ZA (PBMC + ZA) as explained above (Fig. 1). Following 10 days of expansion, untouched γ/δ T cells were isolated from an aliquot of stimulated cells via negative selection using the TCR γ/δ T Cell Isolation Kit (after depletion). Subsequently, a γ/δ and CD3 double staining was employed to flow-cytometrically verify the successful depletion procedure. b + c On day 11, negatively isolated γ/δ T cells (after depletion, grey bars) and the remaining ZA-expanded T cells (black bars) were electroporated with RNA coding for the gp100/A2-specific TCR (b) or with RNA encoding the MCSP-specific CAR (c). T cells electroporated without RNA (mock) served as controls (b + c). Antigen-specific cytokine secretion was determined as described above (Fig. 3). Data represent means ± SEM from 4 [file 12885_2017_3539_MOESM1_ESM.pdf]

TABLE S1. Comparative FACS analysis of different expansion[a] protocols for γδ-TCR, CD3, and CD8 expression

	PBMC + zoledronic acid n = 10		PBMC + OKT3 n = 10		MACS[b] CD8⁺ + OKT3 n = 7		MACS[c] γδ⁺ + OKT3 n = 7	
	**start**	**end**	**start**	**end**	**start**	**end**	**start**	**end**
**$γδ^-$ / $CD3^+$**	59.2 ± 8.14	11.73 ± 9.29	59.2 ± 8.14	74.66 ± 5.85	90.08 ± 5.21	86.43 ± 4	5.32 ± 5.62	2.96 ± 1.89
**$γδ^+$ / $CD3^+$**	11.42 ± 6.99	84.61 ± 10.68	11.42 ± 6.99	18.92 ± 6.05	3.68 ± 3.41	7.91 ± 2.99	84.8 ± 10.08	91.65 ± 5.08
**$γδ^-$ / $CD3^-$**	28.47 ± 8.8	2.67 ± 1.6	28.47 ± 8.8	5.93 ± 2.99	5.93 ± 4.41	5.38 ± 3.86	2.99 ± 2.44	2.31 ± 1.53
**$γδ^+$ / $CD3^-$**	0.91 ± 0.51	0.99 ± 0.78	0.91 ± 0.51	0.5 ± 0.27	0.31 ± 0.28	0.28 ± 0.19	6.89 ± 7.71	3.08 ± 1.93
**$γδ^-$ / $CD8^+$**	26.05 ± 6.86	6.94 ± 6.39	26.05 ± 6.86	70.42 ± 7.98	96.15 ± 3.19	90.62 ± 3.43	1.5 ± 1.53	1.77 ± 1.72
**$γδ^+$ / $CD8^+$**	1.51 ± 0.78	15.2 ± 7.92	1.51 ± 0.78	13.19 ± 5.03	2.16 ± 1.17	7.6 ± 2.92	15.92 ± 6.96	15.87 ± 11.82
**$γδ^-$ / $CD8^-$**	60.67 ± 6.36	7.81 ± 4.29	60.67 ± 6.36	10.14 ± 3.24	0.45 ± 0.22	1.32 ± 0.71	7.05 ± 6.93	3.84 ± 2.08
**$γδ^+$ / $CD8^-$**	11.77 ± 7.23	70.5 ± 17.4	11.77 ± 7.23	6.25 ± 4.87	1.24 ± 2.42	0.46 ± 0.97	75.52 ± 9.94	78.52 ± 13.52

[a]Expansion duration varied from ten to eleven days
[b]$CD8^+$ T cells were extracted from donor-derived PBMC using magnetic-activated cell sorting
[c]$γδ^+$ T cells were extracted from donor-derived PBMC using magnetic-activated cell sorting
[d]Mean ± standard deviation are displayed

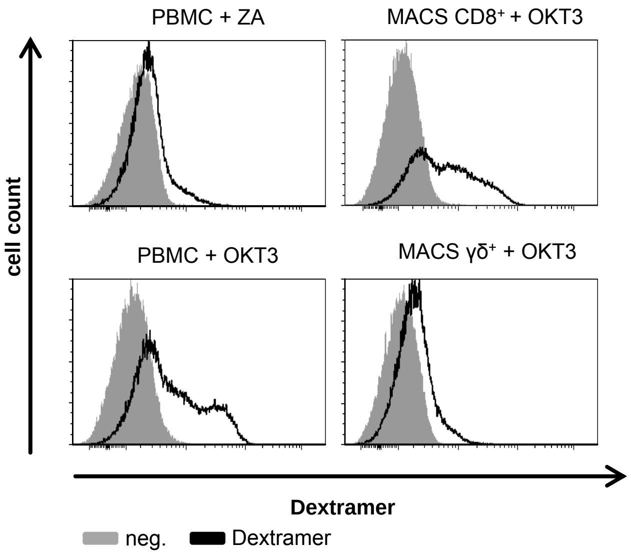

Figure S1: Expression of a gp100/HLA-A2-specific TCR by different T-cell populations after electroporation.

Zoledronic acid (ZA)-activated PBMC, OKT3-activated PBMC, OKT3-stimulated MACS-isolated $CD8^+$ T cells, and OKT3-stimulated MACS-isolated γ/δ T cells were expanded as described in figure 1. After 10-11 days, these cells were electroporated with RNA coding for the gp100/HLA-A2-specific TCR or with RNA encoding the MCSP-specific CAR. After receptor transfer, T cells were rested for one day and subsequently cryopreserved. After thawing TCR expression was detected using a PE-conjugated MHC-Dextramer HLA-A*0201/YLEPGPVTV (black lines). CAR-transfected T cells served as negative controls (neg.; filled grey histograms). Presented histograms are representatives out of three independent experiments.

TABLE S2: P-values corresponding to figure 2 g-j calculated by unpaired Student's t-test

		conditions[#]	time points 4h	24h	48h	72h	96h	120h
**g**	**TCR vs CAR[a]**	**PBMC + ZA** (TCR vs. CAR)	ns	***	*	ns	ns	ns
		**MACS γ/δ + OKT3** (TCR vs. CAR)	***	***	***	***	**	*
**h**	**CAR vs TCR[b]**	**PBMC + ZA** (CAR vs. TCR)	**	***	**	*	*	0.0950
		**MACS γ/δ + OKT3** (CAR vs. TCR)	***	***	***	***	***	***
**i**	**TCR vs CAR**	**PBMC + OKT3** (TCR vs. CAR)	0.0774	***	ns	ns	ns	ns
		**MACS CD8 + OKT3** (TCR vs. CAR)	**	***	**	ns	ns	ns
**j**	**CAR vs TCR**	**PBMC + OKT3** (CAR vs. TCR)	*	***	**	***	***	***
		**MACS CD8 + OKT3** (CAR vs. TCR)	***	**	***	***	***	***

Significance was calculated by unpaired student's t test from 4 independent experiments, * p≤0.05, ** p≤0.01, *** p≤0.001, ns p>0.1, p-values between 0.05 and 0.1 are specified.

[#]Conditions: PBMC + ZA = zoledronic-acid expanded PBMC; PBMC + OKT3 = OKT3-expanded PBMC; MACS CD8 + OKT3 = magnetic-activated cell sorting isolated CD8 T cells expanded with OKT3; γδ + OKT3 = magnetic-activated cell sorting isolated γδ T cells expanded with OKT3; all conditions were either transfected with the gp100 TCR or the MCSP CAR

[a]TCR vs CAR: TCR expression on TCR-transfected T cells against TCR expression on CAR-transfected T cells

[b]CAR vs TCR: CAR expression on CAR-transfected T cells against CAR expression on TCR-transfected T cells

TABLE S3: P-values corresponding to figure 3 calculated by unpaired Student's t-test

	TARGET		CONDITION	IL-2	TNF	IFNγ
				cytokines		
a	Mel526	mock vs TCR	Mel526 **PBMC + ZA** (mock vs. TCR)	ns	ns	ns
			Mel526 **PBMC + OKT3** (mock vs. TCR)	ns	ns	ns
			Mel526 **MACS CD8 + OKT3** (mock vs. TCR)	ns	ns	ns
			Mel526 **MACS γ/δ + OKT3** (mock vs. TCR)	ns	ns	ns
		TCR vs TCR	Mel526 **PBMC + ZA vs. PBMC + OKT3** (TCR vs. TCR)	ns	ns	ns
			Mel526 **PBMC + ZA vs. MACS CD8 + OKT3** (TCR vs. TCR)	ns	ns	ns
			Mel526 **PBMC + ZA vs. MACS γ/δ + OKT3** (TCR vs. TCR)	ns	ns	ns
			Mel526 **PBMC + OKT3 vs. MACS CD8 + OKT3** (TCR vs. TCR)	ns	ns	ns
			Mel526 **PBMC + OKT3 vs. MACS γ/δ + OKT3** (TCR vs. TCR)	ns	ns	ns
			Mel526 **MACS CD8 + OKT3 vs. MACS γ/δ + OKT3** (TCR vs. TCR)	ns	ns	ns
	Mel526 + gp100	mock vs TCR	Mel526+gp100 **PBMC+ ZA** (mock vs. TCR)	0.0713	***	***
			Mel526+gp100 **PBMC+ OKT3** (mock vs. TCR)	***	***	***
			Mel526+gp100 **MACS CD8 + OKT3** (mock vs. TCR)	***	***	***
			Mel526+gp100 **MACS γ/δ + OKT3** (mock vs. TCR)	*	*	***
		TCR vs TCR	Mel526+gp100 **PBMC + ZA vs. PBMC + OKT3** (TCR vs. TCR)	***	**	***
			Mel526+gp100 **PBMC + ZA vs. MACS CD8 + OKT3** (TCR vs. TCR)	***	ns	**
			Mel526+gp100 **PBMC + ZA vs. MACS γ/δ + OKT3** (TCR vs. TCR)	ns	ns	***
			Mel526 +gp100 **PBMC + OKT3 vs. MACS CD8 + OKT3** (TCR vs. TCR)	ns	ns	0.0949
			Mel526+gp100 **PBMC + OKT3 vs. MACS γ/δ + OKT3** (TCR vs. TCR)	***	**	***
			Mel526+gp100 **MACS CD8 + OKT3 vs. MACS γ/δ + OKT3** (TCR vs. TCR)	***	*	***
	A375M	mock vs TCR	A375M **PBMC+ ZA** (mock vs. TCR)	ns	ns	ns
			A375M **PBMC+ OKT3** (mock vs. TCR)	ns	ns	ns
			A375M **MACS CD8 + OKT3** (mock vs. TCR)	ns	ns	ns
			A375M **MACS γ/δ + OKT3** (mock vs. TCR)	ns	ns	ns
		TCR vs TCR	A375M **PBMC + ZA vs. PBMC + OKT3** (TCR vs. TCR)	ns	ns	ns
			A375M **PBMC + ZA vs. MACS CD8 + OKT3** (TCR vs. TCR)	ns	ns	ns
			A375M **PBMC + ZA vs. MACS γ/δ + OKT3** (TCR vs. TCR)	ns	ns	ns
			A375M **PBMC + OKT3 vs. MACS CD8 + OKT3** (TCR vs. TCR)	ns	ns	ns
			A375M **PBMC + OKT3 vs. MACS γ/δ + OKT3** (TCR vs. TCR)	ns	ns	ns
			A375M **MACS CD8 + OKT3 vs. MACS γ/δ + OKT3** (TCR vs. TCR)	ns	ns	ns
	A375M + gp100	mock vs TCR	A375M+gp100 **PBMC+ ZA** (mock vs. TCR)	***	***	***
			A375M+gp100 **PBMC+ OKT3** (mock vs. TCR)	***	***	***
			A375M+gp100 **MACS CD8 + OKT3** (mock vs. TCR)	***	***	***
			A375M+gp100 **MACS γ/δ + OKT3** (mock vs. TCR)	**	***	***
		TCR vs TCR	A375M+gp100 **PBMC + ZA vs. PBMC + OKT3** (TCR vs. TCR)	***	***	***
			A375M+gp100 **PBMC + ZA vs. MACS CD8 + OKT3** (TCR vs. TCR)	***	*	**
			A375M+gp100 **PBMC + ZA vs. MACS γ/δ + OKT3** (TCR vs. TCR)	***	***	***
			A375M+gp100**PBMC + OKT3 vs. MACS CD8 + OKT3** (TCR vs. TCR)	ns	**	ns
			A375M+gp100 **PBMC + OKT3 vs. MACS γ/δ + OKT3** (TCR vs. TCR)	***	***	***
			A375M+gp100 **MACS CD8 + OKT3 vs. MACS γ/δ + OKT3** (TCR vs. TCR)	***	***	***
b	Mel526	mock vs CAR	Mel526 **PBMC+ ZA** (mock vs. CAR)	*	**	***
			Mel526 **PBMC+ OKT3** (mock vs. CAR)	***	***	***
			Mel526 **MACS CD8 + OKT3** (mock vs. CAR)	***	***	***
			Mel526 **MACS γ/δ +O KT3** (mock vs. CAR)	ns	0.0678	**
		CAR vs CAR	Mel526 **PBMC + ZA vs. PBMC + OKT3** (CAR vs. CAR)	***	***	***
			Mel526 **PBMC + ZA vs. MACS CD8 + OKT3** (CAR vs. CAR	***	***	**
			Mel526 **PBMC + ZA vs. MACS γ/δ + OKT3** (CAR vs. CAR)	*	*	**
			Mel526 **PBMC + OKT3 vs. MACS CD8 + OKT3** (CAR vs. CAR)	ns	ns	ns
			Mel526 **PBMC + OKT3 vs. MACS γ/δ + OKT3** (CAR vs. CAR)	***	***	***
			Mel526 **MACS CD8 + OKT3 vs. MACS γ/δ + OKT3** (CAR vs. CAR)	***	***	***
	T2.A1	mock vs CAR	T2.A1 **PBMC + ZA** (mock vs. CAR)	ns	ns	ns
			T2.A1 **PBMC + OKT3** (mock vs. CAR)	ns	ns	ns
			T2.A1 **MACS CD8 + OKT3** (mock vs. CAR)	ns	ns	ns
			T2.A1 **MACS γ/δ + OKT3** (mock vs. CAR)	ns	ns	ns
		CAR vs CAR	T2.A1 **PBMC + ZA vs. PBMC + OKT3** (CAR vs. CAR)	ns	ns	ns
			T2.A1 **PBMC + ZA vs. MACS CD8 + OKT3** (CAR vs. CAR)	ns	ns	ns
			T2.A1 **PBMC + ZA vs. MACS γ/δ + OKT3** (CAR vs. CAR)	ns	ns	ns
			T2.A1 **PBMC + OKT3 vs. MACS CD8 + OKT3** (CAR vs. CAR)	ns	ns	ns
			T2.A1 **PBMC + OKT3 vs. MACS γ/δ + OKT3** (CAR vs. CAR)	ns	ns	ns
			T2.A1 **MACS CD8 + OKT3 vs. MACS γ/δ + OKT3** (CAR vs. CAR)	ns	ns	ns
	A375M	mock vs CAR	A375M **PBMC+ ZA** (mock vs. CAR)	*	***	***
			A375M **PBMC+ OKT3** (mock vs. CAR)	***	***	***
			A375M **MACS CD8 + OKT3** (mock vs. CAR)	***	***	***
			A375M **MACS γ/δ + OKT3** (mock vs. CAR)	*	***	***
		CAR vs CAR	A375M **PBMC + ZA vs. PBMC + OKT3** (CAR vs. CAR)	***	***	***
			A375M **PBMC + ZA vs. MACS CD8 + OKT3** (CAR vs. CAR)	***	***	**
			A375M **PBMC + ZA vs.MACS γ/δ + OKT3** (CAR vs. CAR)	**	***	**
			A375M **PBMC + OKT3 vs. MACS CD8 + OKT3** (CAR vs. CAR)	ns	ns	ns
			A375M **PBMC + OKT3 vs. MACS γ/δ + OKT3** (CAR vs. CAR)	***	***	***
			A375M **MACS CD8 + OKT3 vs. MACS γ/δ + OKT3** (CAR vs. CAR)	***	***	***

Significance was calculated by unpaired student's t test from 7 to 10 independent experiments, * p≤0.05, ** p≤0.01, *** p≤0.001, ns p>0.1, p-values between 0.05 and 0.1 are specified. Conditions: PBMC + ZA = zoledronic-acid expanded PBMC; PBMC + OKT3 = OKT3-expanded PBMC; MACS CD8 + OKT3 = magnetic-activated cell sorting isolated CD8 T cells expanded with OKT3; γδ + OKT3 = magnetic-activated cell sorting isolated γδ T cells expanded with OKT3; all conditions were either electroporated with no RNA (mock) or transfected with the gp100 TCR or the MCSP CAR

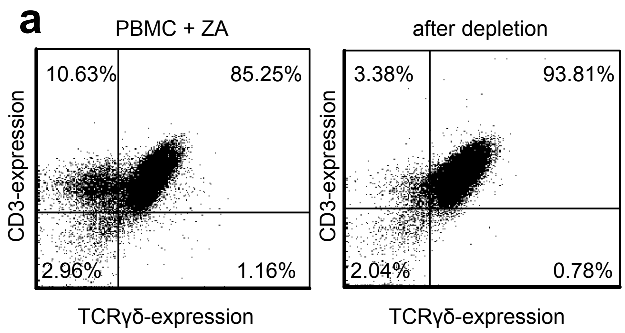

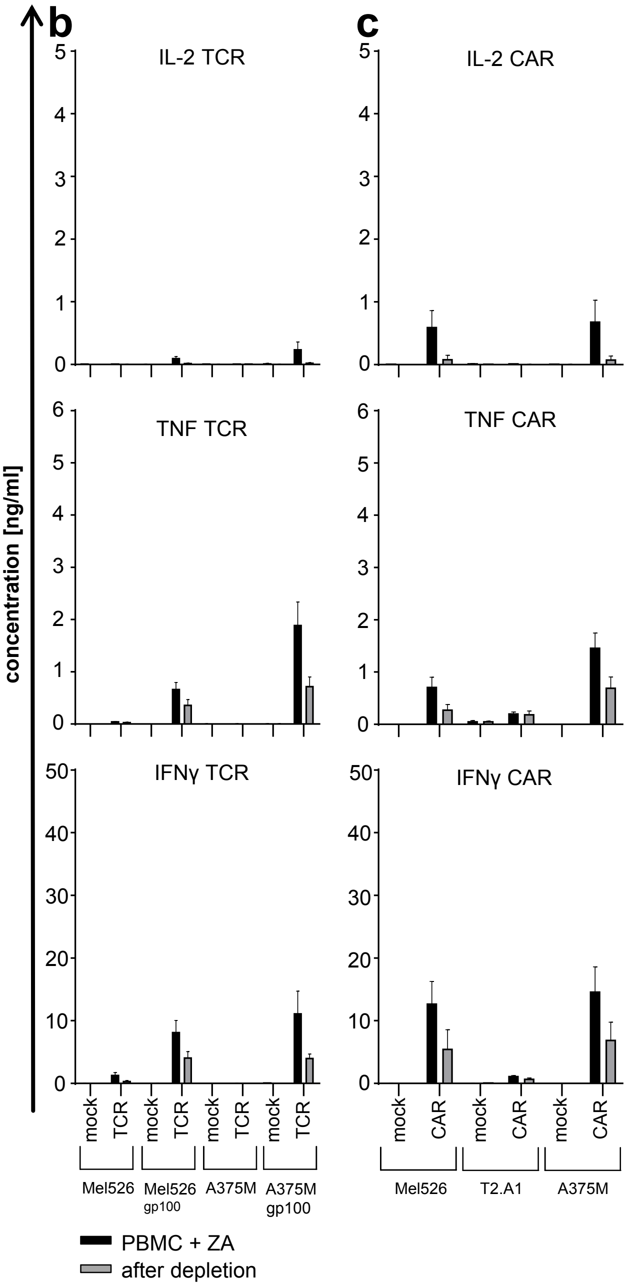

Figure S2: Zoledronic acid-expanded γ/δ T cells retain their cytokine secretory capacity after depletion of γδ⁻ cells.

a Donor-derived PBMC were expanded with ZA (PBMC + ZA) as explained above (Fig. 1). Following 10 days of expansion, untouched γ/δ T cells were isolated from an aliquot of stimulated cells via negative selection using the TCR γ/δ T Cell Isolation Kit (after depletion). Subsequently, a γδ and CD3 double staining was employed to flow-cytometrically verify the successful depletion procedure. **b+c** On day 11, negatively isolated γ/δ T cells (after depletion, grey bars) and the remaining ZA-expanded T cells (black bars) were electroporated with RNA coding for the gp100/A2-specific TCR **(b)** or with RNA encoding the MCSP-specific CAR **(c)**. T cells electroporated without RNA (mock) served as controls **(b+c)**. Antigen-specific cytokine secretion was determined as described above (Fig. 3). Data represent means ± SEM from 4 independent experiments. P values calculated by unpaired Student's t test are presented in additional file 6: Table ST4.

TABLE S4: P-values corresponding to figure S2b and c calculated by unpaired Student's t-test **cytokines**

	TARGET		CONDITION	IL-2	TNF	IFNγ
**b**	**Mel526**	**mock vs TCR**	Mel526 **PBMC + ZA** (mock vs. TCR)	ns	ns	ns
			Mel526 **after depletion** (mock vs. TCR)	ns	ns	ns
		**TCR vs TCR**	Mel526 **PBMC + ZA vs. after depletion** (TCR vs. TCR)	ns	ns	ns
	**Mel526 + gp100**	**mock vs TCR**	Mel526+gp100 **PBMC+ ZA** (mock vs. TCR)	0.0713	***	***
			Mel526+gp100 **after depletion** (mock vs. TCR)	ns	**	**
		**TCR vs TCR**	Mel526+gp100 **PBMC + ZA vs. PBMC + after depletion** (TCR vs. TCR)	ns	ns	*
	**A375M**	**mock vs TCR**	A375M **PBMC+ ZA** (mock vs. TCR)	ns	ns	ns
			A375M **after depletion** (mock vs. TCR)	ns	ns	ns
		**TCR vs TCR**	A375M **PBMC + ZA vs. after depletion** (TCR vs. TCR)	ns	ns	ns
	**A375M + gp100**	**mock vs TCR**	A375M+gp100 **PBMC+ ZA** (mock vs. TCR)	***	***	***
			A375M+gp100 **after depletion** (mock vs. TCR)	0.0682	***	***
		**TCR vs TCR**	A375M+gp100 **PBMC + ZA vs. after depletion** (TCR vs. TCR)	***	***	***
**c**	**Mel526**	**mock vs CAR**	Mel526 **PBMC+ ZA** (mock vs. CAR)	*	**	***
			Mel526 **after depletion** (mock vs. CAR)	ns	0.0794	*
		**CAR vs CAR**	Mel526 **PBMC + ZA vs. after depletion** (CAR vs. CAR)	0.0853	0.0786	0.0603
	**T2.A1**	**mock vs CAR**	T2.A1 **PBMC + ZA** (mock vs. CAR)	ns	ns	ns
			T2.A1 **after depletion** (mock vs. CAR)	ns	ns	ns
		**CAR vs CAR**	T2.A1 **PBMC + ZA vs. after depletion** (CAR vs. CAR)	ns	ns	ns
	**A375M**	**mock vs CAR**	A375M **PBMC+ ZA** (mock vs. CAR)	*	***	***
			A375M **after depletion** (mock vs. CAR)	ns	***	*
		**CAR vs CAR**	A375M **PBMC + ZA vs. after depletion** (CAR vs. CAR)	*	**	*

Significance was calculated by unpaired student's t test from 4 independent experiments, * p ≤0.05, ** p≤0.01, *** p≤0.001, ns p>0.1, p-values between 0.05 and 0.1 are specified.
Conditions: PBMC + ZA = zoledronic-acid expanded PBMC; after depletion = negatively selected γδ T cells from ZA-expanded PBMC; all conditions were either electroporated with no RNA (mock) or transfected with the gp100 TCR or the MCSP CAR

TABLE S5: P-values corresponding to figure 5 a and b calculated by unpaired Student's t-test

	TARGET		CONDITION	effector to target ratio 60 : 1	20 : 1	6 : 1	2 : 1
**a**	**Mel526**	**mock vs TCR**	Mel526 **PBMC + ZA** (mock vs. TCR)	**	ns	ns	ns
			Mel526 **PBMC + OKT3** (mock vs. TCR)	***	*	ns	ns
			Mel526 **MACS CD8 + OKT3** (mock vs. TCR)	***	**	ns	ns
			Mel526 **MACS γ/δ + OKT3** (mock vs. TCR)	0.0790	0.0621	ns	ns
		**mock vs mock**	Mel526 **PBMC + ZA vs. PBMC + OKT3** (mock vs. mock)	ns	ns	ns	ns
			Mel526 **PBMC + ZA vs. MACS CD8 + OKT3** (mock vs. mock)	***	*	*	ns
			Mel526 **PBMC + ZA vs. MACS γ/δ + OKT3** (mock vs. mock)	ns	ns	ns	ns
			Mel526 **PBMC + OKT3 vs. MACS CD8 + OKT3** (mock vs. mock)	**	ns	ns	ns
			Mel526 **PBMC + OKT3 vs. MACS γ/δ + OKT3** (mock vs. mock)	ns	ns	ns	ns
			Mel526 **MACS CD8 + OKT3 vs. MACS γ/δ + OKT3** (mock vs. mock)	**	ns	ns	ns
		**TCR vs TCR**	Mel526 **PBMC + ZA vs. PBMC + OKT3** (TCR vs. TCR)	ns	ns	ns	ns
			Mel526 **PBMC + ZA vs. MACS CD8 + OKT3** (TCR vs. TCR)	ns	ns	ns	ns
			Mel526 **PBMC + ZA vs. MACS γ/δ + OKT3** (TCR vs. TCR)	ns	ns	ns	ns
			Mel526 **PBMC + OKT3 vs. MACS CD8 + OKT3** (TCR vs. TCR)	ns	ns	ns	ns
			Mel526 **PBMC + OKT3 vs. MACS γ/δ + OKT3** (TCR vs. TCR)	ns	ns	ns	ns
			Mel526 **MACS CD8 + OKT3 vs. MACS γ/δ + OKT3** (TCR vs. TCR)	ns	ns	ns	ns
	**A375M**	**mock vs TCR**	A375M **PBMC+ ZA** (mock vs. TCR)	ns	ns	ns	ns
			A375M **PBMC+ OKT3** (mock vs. TCR)	ns	ns	ns	ns
			A375M **MACS CD8 + OKT3** (mock vs. TCR)	ns	ns	ns	ns
			A375M **MACS γ/δ + OKT3** (mock vs. TCR)	ns	ns	ns	ns
		**mock vs mock**	A375M **PBMC + ZA vs. PBMC + OKT3** (mock vs. mock)	*	ns	*	ns
			A375M **PBMC + ZA vs. MACS CD8 + OKT3** (mock vs. mock)	*	0.0693	0.0560	ns
			A375M **PBMC + ZA vs. MACS γ/δ + OKT3** (mock vs. mock)	ns	ns	ns	ns
			A375M **PBMC + OKT3 vs. MACS CD8 + OKT3** (mock vs. mock)	ns	ns	ns	ns
			A375M **PBMC + OKT3 vs. MACS γ/δ + OKT3** (mock vs. mock)	ns	ns	ns	ns
			A375M **MACS CD8 + OKT3 vs. MACS γ/δ + OKT3** (mock vs. mock)	ns	ns	ns	ns
		**TCR vs TCR**	A375M **PBMC + ZA vs. PBMC + OKT3** (TCR vs. TCR)	ns	ns	ns	ns
			A375M **PBMC + ZA vs. MACS CD8 + OKT3** (TCR vs. TCR)	ns	ns	ns	ns
			A375M **PBMC + ZA vs. MACS γ/δ + OKT3** (TCR vs. TCR)	ns	ns	ns	ns
			A375M **PBMC + OKT3 vs. MACS CD8 + OKT3** (TCR vs. TCR)	ns	ns	ns	ns
			A375M **PBMC + OKT3 vs. MACS γ/δ + OKT3** (TCR vs. TCR)	ns	ns	ns	ns
			A375M **MACS CD8 + OKT3 vs. MACS γ/δ + OKT3** (TCR vs. TCR)	*	ns	ns	ns
	**A375M + gp100**	**mock vs TCR**	A375M+gp100 **PBMC+ ZA** (mock vs. TCR)	***	***	ns	ns
			A375M+gp100 **PBMC+ OKT3** (mock vs. TCR)	***	***	**	ns
			A375M+gp100 **MACS CD8 + OKT3** (mock vs. TCR)	***	***	*	ns
			A375M+gp100 **MACS γ/δ + OKT3** (mock vs. TCR)	***	***	0.0896	ns
		**mock vs mock**	A375M+gp100 **PBMC + ZA vs. PBMC + OKT3** (mock vs. mock)	***	*	*	*
			A375M+gp100 **PBMC + ZA vs. MACS CD8 + OKT3** (mock vs. mock)	***	**	*	0.0527
			A375M+gp100 **PBMC + ZA vs. MACS γ/δ + OKT3** (mock vs. mock)	ns	ns	ns	ns
			A375M+gp100**PBMC + OKT3 vs. MACS CD8 + OKT3** (mock vs. mock)	**	ns	ns	ns
			A375M+gp100 **PBMC + OKT3 vs. MACS γ/δ + OKT3** (mock vs. mock)	***	*	0.0709	ns
			A375M+gp100 **MACS CD8 + OKT3 vs. MACS γ/δ + OKT3** (mock vs. mock)	***	**	0.0748	ns
		**TCR vs TCR**	A375M+gp100 **PBMC + ZA vs. PBMC + OKT3** (TCR vs. TCR)	ns	ns	ns	ns
			A375M+gp100 **PBMC + ZA vs. MACS CD8 + OKT3** (TCR vs. TCR)	ns	ns	ns	ns
			A375M+gp100 **PBMC + ZA vs. MACS γ/δ + OKT3** (TCR vs. TCR)	ns	ns	ns	ns
			A375M+gp100**PBMC + OKT3 vs. MACS CD8 + OKT3** (TCR vs. TCR)	ns	ns	ns	ns
			A375M+gp100 **PBMC + OKT3 vs. MACS γ/δ + OKT3** (TCR vs. TCR)	0.0658	ns	ns	ns
			A375M+gp100 **MACS CD8 + OKT3 vs. MACS γ/δ + OKT3** (TCR vs. TCR)	ns	ns	ns	ns
**b**	**Mel526**	**mock vs CAR**	Mel526 **PBMC+ ZA** (mock vs. CAR)	***	**	ns	ns
			Mel526 **PBMC+ OKT3** (mock vs. CAR)	***	***	***	0.0645
			Mel526 **MACS CD8 + OKT3** (mock vs. CAR)	***	***	*	ns
			Mel526 **MACS γ/δ +O KT3** (mock vs. CAR)	*	**	ns	ns
		**CAR vs CAR**	Mel526 **PBMC + ZA vs. PBMC + OKT3** (CAR vs. CAR)	ns	0.0937	ns	ns
			Mel526 **PBMC + ZA vs. MACS CD8 + OKT3** (CAR vs. CAR	ns	ns	ns	ns
			Mel526 **PBMC + ZA vs. MACS γ/δ + OKT3** (CAR vs. CAR)	ns	ns	ns	ns
			Mel526 **PBMC + OKT3 vs. MACS CD8 + OKT3** (CAR vs. CAR)	ns	ns	ns	ns
			Mel526 **PBMC + OKT3 vs. MACS γ/δ + OKT3** (CAR vs. CAR)	ns	0.0652	ns	ns
			Mel526 **MACS CD8 + OKT3 vs. MACS γ/δ + OKT3** (CAR vs. CAR)	ns	ns	ns	ns
	**T2.A1**	**mock vs CAR**	T2.A1 **PBMC + ZA** (mock vs. CAR)	ns	ns	ns	ns
			T2.A1 **PBMC + OKT3** (mock vs. CAR)	ns	ns	0.0880	*
			T2.A1 **MACS CD8 + OKT3** (mock vs. CAR)	**	*	ns	ns
			T2.A1 **MACS γ/δ + OKT3** (mock vs. CAR)	ns	ns	ns	ns
		**mock vs mock**	T2.A1 **PBMC + ZA vs. PBMC + OKT3** (mock vs. mock)	*	*	ns	ns
			T2.A1 **PBMC + ZA vs. MACS CD8 + OKT3** (mock vs. mock)	***	**	*	ns
			T2.A1 **PBMC + ZA vs. MACS γ/δ + OKT3** (mock vs. mock)	ns	ns	ns	ns
			T2.A1 **PBMC + OKT3 vs. MACS CD8 + OKT3** (mock vs. mock)	0.0792	ns	ns	ns
			T2.A1 **PBMC + OKT3 vs. MACS γ/δ + OKT3** (mock vs. mock)	*	ns	ns	ns
			T2.A1 **MACS CD8 + OKT3 vs. MACS γ/δ + OKT3** (mock vs. mock)	**	*	0.0644	0.0734
		**CAR vs CAR**	T2.A1 **PBMC + ZA vs. PBMC + OKT3** (CAR vs. CAR)	ns	ns	ns	ns
			T2.A1 **PBMC + ZA vs. MACS CD8 + OKT3** (CAR vs. CAR)	ns	ns	ns	ns
			T2.A1 **PBMC + ZA vs. MACS γ/δ + OKT3** (CAR vs. CAR)	ns	ns	ns	ns
			T2.A1 **PBMC + OKT3 vs. MACS CD8 + OKT3** (CAR vs. CAR)	ns	ns	ns	0.0919
			T2.A1 **PBMC + OKT3 vs. MACS γ/δ + OKT3** (CAR vs. CAR)	ns	ns	ns	ns
			T2.A1 **MACS CD8 + OKT3 vs. MACS γ/δ + OKT3** (CAR vs. CAR)	ns	ns	ns	ns
	**A375M**	**mock vs CAR**	A375M **PBMC+ ZA** (mock vs. CAR)	***	***	ns	ns
			A375M **PBMC+ OKT3** (mock vs. CAR)	***	***	***	**
			A375M **MACS CD8 + OKT3** (mock vs. CAR)	***	***	*	ns
			A375M **MACS γ/δ + OKT3** (mock vs. CAR)	*	*	ns	ns
		**CAR vs CAR**	A375M **PBMC + ZA vs. PBMC + OKT3** (CAR vs. CAR)	ns	ns	ns	ns
			A375M **PBMC + ZA vs. MACS CD8 + OKT3** (CAR vs. CAR)	ns	ns	ns	ns
			A375M **PBMC + ZA vs.MACS γ/δ + OKT3** (CAR vs. CAR)	ns	ns	ns	ns
			A375M **PBMC + OKT3 vs. MACS CD8 + OKT3** (CAR vs. CAR)	ns	ns	0.0641	ns
			A375M **PBMC + OKT3 vs. MACS γ/δ + OKT3** (CAR vs. CAR)	ns	ns	ns	ns
			A375M **MACS CD8 + OKT3 vs. MACS γ/δ + OKT3** (CAR vs. CAR)	ns	ns	ns	ns

Significance was calculated by unpaired student's t test from 4 to 7 independent experiments, * p≤0.05, ** p≤0.01, *** p≤0.001, ns p>0.1, p-values between 0.05 and 0.1 are specified.
Conditions: PBMC + ZA = zoledronic-acid expanded PBMC; PBMC + OKT3 = OKT3-expanded PBMC; MACS CD8 + OKT3 = magnetic-activated cell sorting isolated CD8 T cells expanded with OKT3; γδ + OKT3 = magnetic-activated cell sorting isolated γδ T cells expanded with OKT3; all conditions were either electroporated with no RNA (mock) or transfected with the gp100 TCR or the MCSP CAR

TABLE S6: P-values corresponding to figure 5 c and d calculated by unpaired Student's t-test **effector to target ratio**

	TARGET		CONDITION	60 : 1	20 : 1	6 : 1	2 : 1
c	Mel526	mock vs TCR	Mel526 **PBMC + ZA** (mock vs. TCR)	**	ns	ns	ns
			Mel526 **after depletion** (mock vs. TCR)	ns	ns	ns	ns
		mock vs mock	Mel526 **PBMC + ZA vs. after depletion** (mock vs. mock)	ns	ns	ns	ns
		TCR vs TCR	Mel526 **PBMC + ZA vs. after depletion** (TCR vs. TCR)	ns	ns	ns	ns
	A375M	mock vs TCR	A375M **PBMC+ ZA** (mock vs. TCR)	ns	ns	ns	ns
			A375M **PBMC+ after depletion** (mock vs. TCR)	ns	ns	ns	ns
		mock vs mock	A375M **PBMC + ZA vs. after depletion** (mock vs. mock)	ns	ns	ns	ns
		TCR vs TCR	A375M **PBMC + ZA vs. after depletion** (TCR vs. TCR)	ns	ns	ns	ns
	A375M + gp100	mock vs TCR	A375M+gp100 **PBMC+ ZA** (mock vs. TCR)	***	***	ns	ns
			A375M+gp100 **after depletion** (mock vs. TCR)	***	0.0587	ns	ns
		mock vs mock	A375M+gp100 **PBMC + ZA vs. after depletion** (mock vs. mock)	ns	ns	ns	ns
		TCR vs TCR	A375M+gp100 **PBMC + ZA vs. after depletion** (TCR vs. TCR)	ns	ns	ns	ns
d	Mel526	mock vs CAR	Mel526 **PBMC+ ZA** (mock vs. CAR)	***	**	ns	ns
			Mel526 **PBMC+ after depletion** (mock vs. CAR)	ns	0.0707	ns	0.0645
		CAR vs CAR	Mel526 **PBMC + ZA vs. after depletion** (CAR vs. CAR)	ns	ns	ns	ns
	T2.A1	mock vs CAR	T2.A1 **PBMC + ZA** (mock vs. CAR)	ns	ns	ns	ns
			T2.A1 **PBMC + after depletion** (mock vs. CAR)	ns	ns	ns	ns
		mock vs mock	T2.A1 **PBMC + ZA vs. after depletion** (mock vs. mock)	ns	ns	ns	ns
		CAR vs CAR	T2.A1 **PBMC + ZA vs. after depletion** (CAR vs. CAR)	ns	ns	ns	ns
	A375M	mock vs CAR	A375M **PBMC+ ZA** (mock vs. CAR)	***	***	ns	ns
			A375M **PBMC+ after depletion** (mock vs. CAR)	ns	ns	ns	ns
		CAR vs CAR	A375M **PBMC + ZA vs. after depletion** (CAR vs. CAR)	*	ns	ns	ns

Significance was calculated by unpaired student's t test from 3 independent experiments, * p≤0.05, ** p≤0.01, *** p≤0.001, ns p>0.1, p-values between 0.05 and 0.1 are specified.
Conditions: PBMC + ZA = zoledronic-acid expanded PBMC; after depletion = negatively selected γδ T cells from ZA-expanded PBMC; all conditions were either electroporated with no RNA (mock) or transfected with the gp100 TCR or the MCSP CAR

TABLE S7: P-values corresponding to figure 6 calculated by unpaired Student's t-test

TARGET		CONDITION	effector to target ratio 20 : 1	6 : 1	2 : 1
Daudi	mock vs TCR	Mel526 **PBMC + ZA** (mock vs. TCR)	ns	ns	ns
		Mel526 **PBMC + OKT3** (mock vs. TCR)	ns	ns	ns
		Mel526 **MACS CD8 + OKT3** (mock vs. TCR)	ns	ns	ns
		Mel526 **MACS γ/δ + OKT3** (mock vs. TCR)	ns	ns	ns
	mock vs mock	Mel526 **PBMC + ZA vs. PBMC + OKT3** (mock vs. mock)	*	*	*
		Mel526 **PBMC + ZA vs. MACS CD8 + OKT3** (mock vs. mock)	**	**	0.0667
		Mel526 **PBMC + ZA vs. MACS γ/δ + OKT3** (mock vs. mock)	ns	ns	ns
		Mel526 **PBMC + OKT3 vs. MACS CD8 + OKT3** (mock vs. mock)	ns	ns	ns
		Mel526 **PBMC + OKT3 vs. MACS γ/δ + OKT3** (mock vs. mock)	ns	ns	ns
		Mel526 **MACS CD8 + OKT3 vs. MACS γ/δ + OKT3** (mock vs. mock)	0.0860	0.0778	ns
	TCR vs TCR	Mel526 **PBMC + ZA vs. PBMC + OKT3** (TCR vs. TCR)	0.0602	0.0857	ns
		Mel526 **PBMC + ZA vs. MACS CD8 + OKT3** (TCR vs. TCR)	**	*	0.0697
		Mel526 **PBMC + ZA vs. MACS γ/δ + OKT3** (TCR vs. TCR)	ns	ns	ns
		Mel526 **PBMC + OKT3 vs. MACS CD8 + OKT3** (TCR vs. TCR)	ns	ns	ns
		Mel526 **PBMC + OKT3 vs. MACS γ/δ + OKT3** (TCR vs. TCR)	0.0646	ns	ns
		Mel526 **MACS CD8 + OKT3 vs. MACS γ/δ + OKT3** (TCR vs. TCR)	*	ns	ns
	mock vs CAR	Mel526 **PBMC+ ZA** (mock vs. CAR)	ns	ns	ns
		Mel526 **PBMC+ OKT3** (mock vs. CAR)	ns	ns	ns
		Mel526 **MACS CD8 + OKT3** (mock vs. CAR)	ns	ns	ns
		Mel526 **MACS γ/δ +O KT3** (mock vs. CAR)	ns	ns	ns
	CAR vs CAR	Mel526 **PBMC + ZA vs. PBMC + OKT3** (CAR vs. CAR)	ns	ns	ns
		Mel526 **PBMC + ZA vs. MACS CD8 + OKT3** (CAR vs. CAR	**	**	ns
		Mel526 **PBMC + ZA vs. MACS γ/δ + OKT3** (CAR vs. CAR)	ns	ns	ns
		Mel526 **PBMC + OKT3 vs. MACS CD8 + OKT3** (CAR vs. CAR)	0.0659	ns	ns
		Mel526 **PBMC + OKT3 vs. MACS γ/δ + OKT3** (CAR vs. CAR)	ns	ns	ns
		Mel526 **MACS CD8 + OKT3 vs. MACS γ/δ + OKT3** (CAR vs. CAR)	**	*	0.0670

Significance was calculated by unpaired student's t test from 3 to 5 independent experiments, * p≤0.05, ** p≤0.01, *** p≤0.001, ns p>0.1, p-values between 0.05 and 0.1 are specified.
Conditions: PBMC + ZA = zoledronic-acid expanded PBMC; PBMC + OKT3 = OKT3-expanded PBMC; MACS CD8 + OKT3 = magnetic-activated cell sorting isolated CD8 T cells expanded with OKT3; γδ + OKT3 = magnetic-activated cell sorting isolated γδ T cells expanded with OKT3; all conditions were either electroporated with no RNA (mock) or transfected with the gp10 TCR or the MCSP CAR
